# Oral Microalgae‐Based Biosystem to Enhance Irreversible Electroporation Immunotherapy in Hepatocellular Carcinoma

**DOI:** 10.1002/advs.202409381

**Published:** 2025-01-28

**Authors:** Cheng Zeng, Shiyuan Hua, Jiayu Zhou, Tangye Zeng, Jianke Chen, Lijian Su, Angfeng Jiang, Min Zhou, Zhe Tang

**Affiliations:** ^1^ Department of Surgery Center for Cancer Medicine the Fourth Affiliated Hospital of School of Medicine International School of Medicine International Institutes of Medicine Zhejiang University Yiwu 322000 China; ^2^ Zhejiang University‐University of Edinburgh Institute (ZJU‐UoE Institute) Zhejiang University Haining 314400 China; ^3^ Department of Surgery The Second Affiliated Hospital Zhejiang University School of Medicine Hangzhou 310000 China; ^4^ Zhejiang Key Laboratory of Precision Diagnosis and Treatment for Lung Cancer the Fourth Affiliated Hospital, Zhejiang University School of Medicine Yiwu 322000 China; ^5^ Institute of Translational Medicine Zhejiang University Hangzhou 310029 China; ^6^ Zhejiang University‐Ordos City Etuoke Banner Joint Research Center Zhejiang University Haining 314400 China; ^7^ The National Key Laboratory of Biobased Transportation Fuel Technology Zhejiang University Hangzhou 310027 China; ^8^ School of Medicine Shihezi University Shihezi Xinjiang 832002 China

**Keywords:** *Chlorella vulgaris* (*C. vulgaris*), combination therapy, hepatocellular carcinoma, irreversible electroporation, polydopamine

## Abstract

Irreversible electroporation (IRE) is a novel local tumor ablation technique that can potentially stimulate immune responses. However, IRE alone cannot effectively activate the immune system or prevent distant metastases. Therefore, this study utilized the biocompatibility of *Chlorella vulgaris* (*C. vulgaris*) and polydopamine (PDA) adhesive properties to encapsulate a PD‐1 inhibitor (PI). The PDA coating protects the drug from degradation by stomach acid and enhances its intestinal absorption. This carrier demonstrates excellent in vivo drug release control and biodistribution, significantly increasing the oral bioavailability of PI. Combining IRE with this natural carrier significantly improves the therapeutic efficacy, which increases the local drug concentration and activates the immune system. This system demonstrates significantly improved therapeutic efficacy against local tumors compared with PI or IRE alone and significantly reduces PI‐associated side effects. A convenient oral delivery system is developed using this readily available natural micro‐carrier that not only improves the therapeutic effect of IRE but also mitigates its adverse effects, indicating significant potential for clinical applications. This discovery offers a new strategy for hepatocellular carcinoma treatment with the potential to improve patient outcomes.

## Graphical abstract

This study presents an innovative oral microalgae‐based biosystem to inhibit tumor growth, reduce PD‐1 expression, and activate immune responses of hepatocellular carcinoma. When combined with enhanced irreversible electroporation treatment, it demonstrates a synergistic effect that effectively reduces the tumor burden and enhances the tumor immune mechanism, thereby opening a new avenue for treating hepatocellular carcinoma.

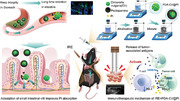

## 1 Introduction

Hepatocellular carcinoma (HCC) has recently become a major global health concern, with 906,000 new cases and 830,000 deaths reported in 2020. Despite advances in medical science, the incidence of HCC persists, especially in China, owing to an aging population.^[^ ^1^, ^2^, ^3^, ^4^ ^]^ More than 50% of patients experience postoperative recurrence, with a 5‐year survival rate of 18%. The high recurrence rate and risk of liver failure associated with re‐resection underscore the urgent need for innovative treatments for HCC.^[^ ^5^, ^6^, ^7^ ^]^

Currently, HCC is managed through a combination of various non‐surgical and surgical approaches. These include transcatheter hepatic artery chemoembolization, radiofrequency ablation, microwave ablation, radioactive particle placement, radiotherapy, chemotherapy, and the relatively new nanoknife ablation technique, which was recently introduced to the clinic.^[^ ^8^, ^9^ ^]^ Nanoknife ablation, also known as irreversible electroporation (IRE), is a physical ablation method that induces apoptosis in cancer cells. This process creates an irreversible nanoscale opening in the cancer cell membrane, independent of thermal impact, without causing damage to vital structures, such as blood vessels or bile ducts within the ablation region. The desired ablation size is ≈50–70 cm^3^, which effectively extends patient survival by simultaneously adjusting the pulse intensity and duration, making it suitable for treating localized HCC.^[^ ^10^, ^11^ ^]^ Tumor recurrence after IRE treatment is caused by tumor cell survival. This is because the electric field intensity within the treatment area is highest at the treatment center (closest to the electrodes) and decreases with increasing distance from the center. Therefore, the tumor edges exhibit the weakest electric field intensity. When the electric field intensity is below 1000 V cm^−1^, the process is considered reversible electroporation (RE) rather than IRE, which allows the cell membrane to reseal after electroporation and enables cell survival.^[^ ^12^ ^]^ Furthermore, residual tumor tissue surviving at the tumor margins after IRE exhibits elevated expression of epithelial cell adhesion molecules, exacerbating invasiveness and accelerating growth, thereby increasing the difficulty and complexity of subsequent treatments.^[^ ^13^ ^]^ Moreover, recurring tumor tissues demonstrate high expression of epithelial cell adhesion factor, which enhances invasiveness, accelerates growth, and increases the likelihood of recurrence.

Liver ablation techniques, including radiofrequency and microwaves, are widely used to treat HCC. Thermal ablation causes coagulative necrosis; however, incomplete ablation may occur with radiofrequency ablation when the tumor is >3 cm and adjacent to blood vessels or the hepatic portal, significantly reducing the safety and efficacy.^[^ ^14^ ^]^ Percutaneous ethanol injection remains a viable treatment for HCC where radiofrequency ablation is unsuitable, such as near the gallbladder, stomach, colon, or other viscera. Microwave ablation has emerged as a promising technique, achieving encouraging response rates in tumors 3–5 cm in size and those adjacent to vessels and the gallbladder. It requires fewer sessions and provides overall survival comparable to radiofrequency ablation.^[^ ^5^, ^15^ ^]^ However, the heat‐sink effect does not affect the IRE or damage the adjacent structures. Accordingly, it may solve the problem of liver tumor ablation at specific locations. It has the advantages of being minimally invasive and has a short treatment time, safety, and reliability. Cryoablation, another alternative, requires liquid gases, such as argon or nitrogen, and induces local freezing of tissues, resulting in a combination of necrotic and delayed apoptotic cell death. Theoretically, IRE has a distinct advantage in treating para‐vascular and para‐biliary tumors.^[^ ^16^, ^17^ ^]^ However, it also has some drawbacks, including hypothermal damage to the neighboring blood vessels, the need for several freeze–thaw cycles, and the need for real‐time monitoring of the ice ball for precise ablation.^[^ ^18^ ^]^

Rapid advancements in tumor immunotherapy and a growing understanding of the immune microenvironment of HCC have shifted the focus toward novel systemic treatments, including immune checkpoint inhibitors, such as programmed death receptor 1 and its ligand (PD‐1/PD‐L1) inhibitors, and cytotoxic *T*‐lymphocyte‐associated antigen 4 inhibitors.^[^ ^19^ ^]^ Nevertheless, solitary immunotherapy encounters challenges such as limited drug efficacy, elevated autoimmune toxicity, and increased vulnerability to drug resistance due to the unique tumor microenvironment in HCC.^[^ ^20^, ^21^ ^]^ Numerous recent reports have indicated that IRE can enhance cell membrane permeability, trigger immunogenic cell death, expose various inherent tumor antigens, facilitate immune cell infiltration, and consequently elicit a specific level of anti‐cancer immune response.^[^ ^22^ ^]^ The combined application of IRE and immunotherapy has emerged as a prominent area of research aimed at decreasing patient relapse and extending survival in HCC treatment.

Monoclonal antibody biomolecule medications have significant potential for treating major illnesses such as tumors, diabetes, and infectious diseases, owing to their potent pharmacological activity and specific targeting. However, their clinical use is limited to injectable forms. Moreover, these drugs often exhibit a short half‐life, high toxicity, and challenges in patient compliance during long‐term drug administration.^[^ ^23^, ^24^ ^]^ We aim to develop a novel drug delivery system that is safer than the traditional approaches. Oral administration can reduce the risk of healthcare‐associated infections related to intravenous administration. The fundamental principle of oral immune checkpoint inhibitors involves drug entry into the intestine and absorption by intestinal epithelial cells, followed by entry into circulation to reach the tumor site.^[^ ^25^ ^]^ Developing effective oral formulations based on microalgal technology has the potential to revolutionize the occurrence and progression of diseases, providing patients with a more convenient and user‐friendly alternative to traditional drug administration methods. Oral formulations offer numerous advantages, including fewer side effects, better patient compliance, convenience, and non‐invasiveness.^[^ ^26^, ^27^ ^]^ Our findings indicate that oral formulations can activate the immune system after IRE and offer superior biosafety compared with intravenous injections. Yang et al. constructed a drug delivery carrier for oral administration of PD‐L1 inhibitors to enhance immunotherapy efficacy.^[^ ^28^ ^]^ Sasikumar et al. reported new oral immune checkpoint inhibitors (CA‐170 and YPD‐30) and confirmed their ability to promote CD8 cell activation.^[^ ^29^ ^]^ These results support the rationale for using oral immune checkpoint inhibitors and that combining IRE with oral polydopamine‐Chlorella vulgaris (PDA‐CV)@PI holds promise as a more effective treatment option for patients. Moreover, oral dosage forms have become a focal point for investigating biomacromolecule drug delivery systems because of their straightforward, safe, and non‐invasive nature.^[^ ^30^ ^]^ However, oral administration of these hydrophilic biomolecules is challenging because of their limited absorption by lipophilic biofilms. They are easily degraded by gastric acid and enzymes and are susceptible to the hepatic first‐pass effect, resulting in reduced activity or inactivation, low bioavailability, and short half‐life.^[^ ^31^, ^32^ ^]^

Advancements in oral drug delivery materials and molecular biology have led to the development of numerous innovative treatment methods, including polymeric nanoparticles,^[^ ^33^ ^]^ liposomes,^[^ ^34^ ^]^ micelles,^[^ ^35^ ^]^ and microspheres,^[^ ^36^ ^]^ which transport bioactive compounds such as proteins, peptides, and nucleic acids. Various methods have been developed for HCC management; however, significant challenges remain, including technical difficulties, expensive materials, and inefficient manufacturing associated with these intricate synthesis procedures. Moreover, the clinical application of these materials, produced through chemical synthesis and engineering, is significantly hindered by their limited natural degradation, lack of durability, and potential damage to living organisms.^[^ ^37^, ^38^ ^]^ Therefore, developing an effective oral drug delivery system is essential. In this regard, *Chlorella vulgaris* (CV), a natural microalga, offers a promising solution.

CV is suitable for human consumption due to its high nutrient content and immune‐boosting properties.^[^ ^39^ ^]^ Its negative surface charge allows electrostatic adsorption of positively charged drugs, thereby enhancing drug loading.^[^ ^40^ ^]^ Previous studies have demonstrated that incorporating drugs such as amphotericin into microalgae, such as *Spirulina*, can improve treatment outcomes for conditions including lung metastasis from breast cancer^[^ ^41^ ^]^ and intestinal diseases.^[^ ^42^ ^]^ The potential of CV as a drug carrier is further supported by its ability to mitigate tumor hypoxia when coated with a calcium phosphate layer and administered intravenously.^[^ ^43^ ^]^ The PDA is a versatile biomaterial that improves the stability and absorption of monoclonal antibodies in the gastrointestinal tract.^[^ ^44^ ^]^ It forms a protective coating through oxidative self‐polymerization of dopamine, enhancing biocompatibility and adhesion.^[^ ^45^ ^]^ It can improve the absorption of orally administered proteins and peptides by adhering to the intestinal mucosa, thereby increasing drug bioavailability.^[^ ^46^ ^]^ When used as a shell coating for CV, PDA can enhance the intestinal retention and absorption of monoclonal antibodies,^[^ ^47^ ^]^ rendering it an ideal material for developing an oral microcapsule delivery system. This approach combines the benefits of a natural biological carrier with a synthetic coating, offering a cost‐effective, eco‐friendly, and easily synthesized solution for oral drug delivery.

**Scheme 1:**
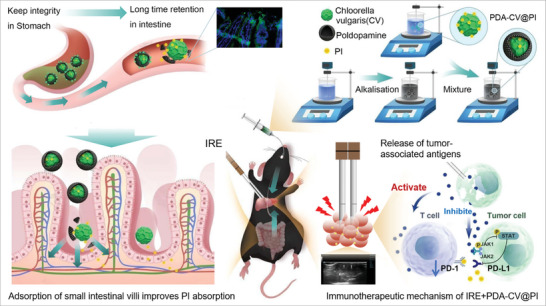
Schematic of PDA‐CV@PI for HCC. In vivo experiments demonstrated that PDA‐CV@PI significantly inhibited tumor growth, reduced PD‐1 expression, and activated immune responses. When combined with IRE, it demonstrated a synergistic effect that effectively reduced the tumor burden and enhanced the tumor immune mechanism, thereby opening a new avenue for treating HCC.

In this study, we developed a novel drug delivery system for PDA‐CV@PD‐1 inhibitors (PI), combining the protective and adhesive properties of PDA with the biocompatibility and drug‐loading capacity of CV microalgae. This novel formulation of PI can be administered orally with fewer side effects than intravenous administration, and there were no significant differences in the therapeutic effects. Our findings also demonstrated that oral administration promoted an immune response after IRE. Therefore, we proposed that oral administration of the immune checkpoint inhibitor overcomes the “Reversible perforation” limitations of IRE. Simultaneously, this composite system not only enhanced the stability of the PI in the gastric acid environment but also improved its adhesion and permeability in the small intestinal mucosa through surface modification of PDA, which prolonged the residence time of the drug in the digestive tract. When combined with IRE technology, this system demonstrated a synergistic effect, effectively reducing the tumor burden and enhancing the tumor immune mechanism. This system aims to improve the oral bioavailability and therapeutic efficacy of PI, providing a novel strategy for HCC immunotherapy through a convenient oral administration approach (**Scheme** **1**).

## 2 Results

### 2.1 Preparation and Characterization of PDA‐CV@PI

**Figure 1:**
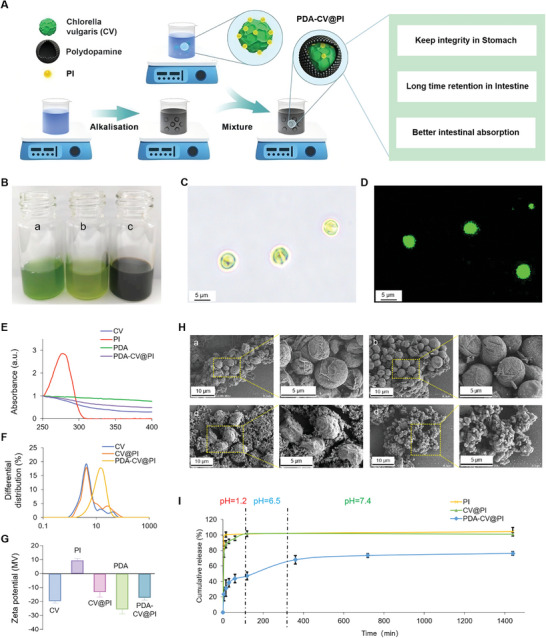
Synthesis and characterization of PDA‐CV@PI. A) Schematic diagram of PDA‐CV@PI formation; B) appearance of the finished product (a: CV, b: CV@PI, and c: PDA‐CV@PI); C) micrograph of CV; D) fluorescence of CV; E) UV absorbance; F) particle size change; G) zeta potential variation plots; H) SEM images (a: CV, b: CV@PI, c: PDA‐CV(1:1)@PI, and d: PDA‐CV(10:1)@PI); and I) in vitro release degree variation.

**Figure** **1**A illustrates the preparation of PDA‐encapsulated CV@PI, where PI was loaded onto CV through electrostatic adsorption and subsequently modified with PDA. The CV was green and spherical, as evidenced by the visual appearance of CV, CV@PI, and PDA‐encapsulated CV@PI (Figure 1B‐a–c), which became darker after the PDA coating. Figure 1C,D illustrates the morphology of the protein and Chlorella under a microscope, along with their fluorescence properties. Figure 1E illustrates the UV absorbance of CV and its constituent components, indicating that CV absorption varied with the different treatments. The intensity‐average hydrodynamic diameter of CV@PI was 4.52 ± 0.37 µm, while that of PDA‐encapsulated CV@PI was 19.27 ± 1.55 µm, with a zeta‐potential of 11.91 ± 2.43 mV in acidic environment (Figure 1F,G). The observed change in the hydrodynamic diameter of the PDA‐encapsulated CV@PI may be attributed to PDA‐induced aggregation. Subsequently, the drug adsorption rate of the PDA‐encapsulated CV@PI was evaluated, revealing that the adsorption rate reached a maximum of 91%. Scanning electron microscopy further confirmed that CV@PI exhibited spherical morphology, uniform dispersion, and comparable size. Furthermore, the surface of the inhibitor displayed localized protrusions and irregular textures (Figure 1H). The PDA‐encapsulated CV@PI exhibited a smoother surface than CV@PI, with distinct small spherical PDA particles encapsulated on its exterior. The optimum mass ratio of PDA to CV was 10:1, which resulted in the best encapsulation effect of CV@PI. Moreover, the peaks at 1077, 1438, 1601, and 3224 cm^−1^ in the PDA spectrum correspond to the stretching vibrations of the C─O, C─N, C═C, and O─H bonds, respectively (Figure S1, Supporting Information). The consistency of the PDA structure with the literature indicates its successful preparation.^[^ ^48^ ^]^ Finally, after encapsulating CV@PI with PDA, the intensities of various chemical absorption peaks in PDA‐CV@PI were significantly reduced. The peaks corresponding to the O─H, C─H, C═C, and C─O bonds were prominent, indicating that PDA successfully encapsulated CV@PI.

Drug release is an important characteristic of nanoparticles. The accumulated release behaviors of CV@PI and PDA‐coated CV@PI were investigated. The time‐dependent cumulative release of the oral groups treated with CV@PI and PDA‐coated CV@PI was fitted using four equations (Table S1, Supporting Information). The group treated with CV@PI exhibited a rapid initial release, with over 90% of the drug released within 2 h (Figure 1I, Table S1, Supporting Information), after which the maximum release was achieved. Conversely, CV@PI exhibited ≈43.2% drug release within 2 h, and the degree of drug release exceeded 95% after 12 h. However, the PDA‐coated CV demonstrated a significantly delayed release effect compared to the uncoated CV. The in vitro release of the CV@PI group followed Weibull's kinetic equation (*r^2^ *= 0.936) and conformed to the first‐order kinetic equation (*r^2^ *= 0.9834), with T25, T50, Td, and T80 values of 6.8, 132.5, 337.2, and 486.7 min, respectively. These values were significantly different (*P *< 0.05), indicating the presence of a slow‐release effect in the PDA‐coated CV@PI group.

### 2.2 Cellular Toxicity of PDA‐Encapsulated CV

**Figure 2:**
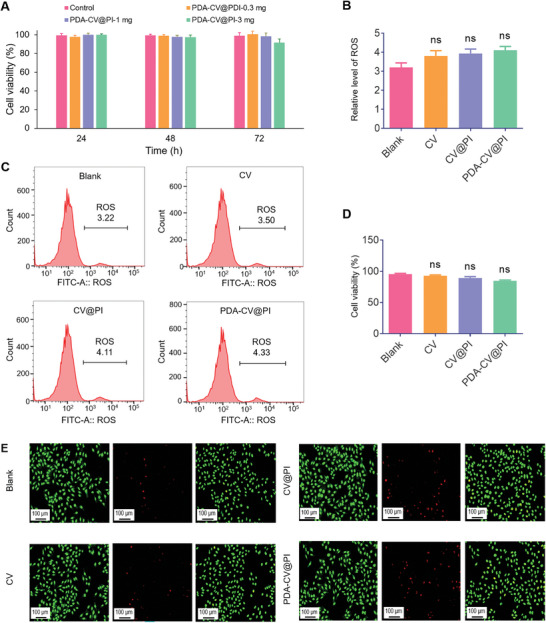
Evaluation of cellular toxicity of PDA‐CV@PI. A) Cell viability; B) relative levels of ROS; C) intracellular ROS fluctuations; D) positive cell rate of calcein‐AM; and E) dual staining of live/dead cells. Values are expressed as mean ± SD. (versus the control group, ns: *p* > 0.05, *: *p* < 0.05, **: *p* < 0.01, and ***: *p* < 0.001 determined by Student's *t*‐test).

As illustrated in **Figure** **2**A, compared with the control group, the cell viability of the other groups was >90% (*p* > 0.05) within 72 h, suggesting a mild toxic effect on Hepa1‐6 cells. There was a slight increase in intracellular reactive oxygen species (ROS) levels in the PDA‐CV@PI group compared to those in the control group (*p* > 0.05, Figure 2B,C). Live/dead cell double staining was used to further validate the toxicity of the microalgal spheres. As depicted in Figure 2D,E, the CV and CV@PI groups demonstrated slight cytotoxicity compared with the control group. However, in the CV group containing PI enclosed with PDA, there was a limited level of cell detachment and a decreased presence of cells stained red. This discovery aligns with the outcomes of ROS flow cytometry evaluation. These results suggest that PDA‐CV@PI has mild toxicity to cells and good biocompatibility.

### 2.3 In Vivo Distribution and Pharmacokinetic Studies

**Figure 3:**
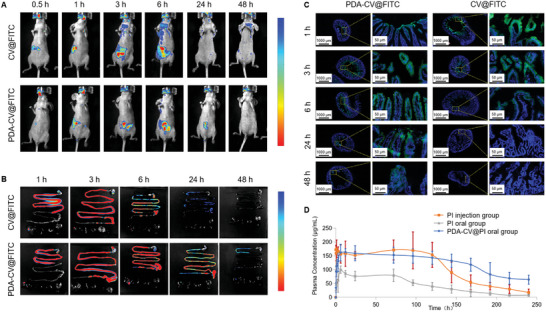
In vivo distribution and pharmacokinetic study. A) Fluorescence images of the body and gastrointestinal tract of mice at 0.5, 1, 3, 6, 24, and 48 h after oral administration of CV@FITC or PDA‐CV@FITC (with an equal amount of FITC). FITC channel: Ex, 445–490 nm, Em, 515–575 nm. The green dotted circle indicates the analyzed area for the fluorescence intensity quantification; B) fluorescence images of the gastrointestinal tract and major organs (from left to right: liver, heart, spleen, lungs, and kidneys) of CV@FITC and PDA‐CV@FITC at different time points after gavage; C) fluorescence images of CV@FITC and PDA‐CV@FITC on the gastrointestinal mucosa at various time points; and D) drug concentration–time curve of PI injection, PI oral, and PDA‐CV@PI oral groups.

PI is a key immunosuppressive molecule and a member of the immunoglobulin superfamily. It can kill and treat exogenous pathogenic microorganisms and regulate autoimmune diseases through targeted stimulation, making it crucial for organ transplantation survival. However, PI lacks fluorescence properties, which makes its detection challenging. When fluorescein isothiocyanate (FITC) reacts with antibody proteins in an alkaline solution, it forms FITC‐protein conjugates, also called fluorescent antibodies. By substituting FITC for PI, PDA‐CV acquires fluorescence. Therefore, FITC can be used to simulate the PI distribution in vivo. **Figure** **3**A illustrates fluorescence imaging of mice and isolated gastrointestinal tracts in the PDA‐CV@FITC and CV@FITC groups at different time intervals. The results indicated that the fluorescence signals in the digestive system of the CV@FITC group gradually decreased over time, with intense red fluorescence signals after 1 h and faint blue or no signals after 24 h. In the cecum and colon, a negligible fluorescence signal was observed after 1 h, which transitioned to a strong red fluorescence signal before weakening. In the PDA‐CV@FITC group, the FITC marker penetrated the intestine within 1 h and reached the cecum and colon by 3 h, indicating the widespread distribution of PDA‐CV@FITC throughout the gastrointestinal tract at 3 h. Subsequently, the red fluorescence signals in the stomach and small intestine gradually decreased.

In contrast, the cecum and colon exhibited strong red fluorescence signals at 6 h, followed by a decline in the red fluorescent signals at 24 h. After 24 h, the red fluorescence signals in the cecum and colon decreased, and the fluorescence signals in all parts of the gastrointestinal tract reached their lowest levels after 48 h. Notably, some of the material may adhere to the skin near the mouth during gavage and remain unmetabolized, as it does not reach the gastrointestinal tract, resulting in a strong signal near the mouth. Conversely, fluorescence imaging interferes with the background signals. Although the skin signals of nude mice were weak, there were still some interference signals in the teeth and mouth. Therefore, a strong fluorescence signal was retained in the oral cavity of mice after 48 h. However, ex vivo fluorescence imaging provided more accurate results.

The PDA‐CV@FITC group primarily accumulated in the gastrointestinal tract after 24 h (Figure 3B,C). Although no fluorescence signals were observed in the liver, heart, spleen, lungs, or kidneys, the gastrointestinal tract still exhibited strong fluorescence signals, implying that PDA‐CV@FITC prolonged intestinal retention. Furthermore, FITC was continuously released and was completely degraded. Conversely, the PDA shells improved intestinal absorption. PDA‐CV@FITC exhibited significantly stronger fluorescence in the intestinal villi and submucosa of the duodenum than CV@FITC. This implies that drug absorption can be significantly enhanced by the PDA coating and CV, resulting in improved intestinal penetration. Studies have indicated that PDA shells can engage with mucins in the intestinal mucus. Therefore, PDA and CV can mutually assist each other in improving intestinal distribution, making PDA‐CV@drug a rational and effective combination.

The PI intravenous, PI oral, and PDA‐CV@PI groups exhibited adherence to the two‐compartment model for fitting plasma concentration‐time curves (with minimal Akaike's Information Criteria values), as indicated in Table S2 (Supporting Information) and Figure 3D. The results demonstrated that the absorption was enhanced, and the elimination was delayed owing to the PDA‐modified CV. The PK parameters displayed in Table S2 (Supporting Information) reveal that the *C*_max_ of the group treated with PDA‐CV@PI was 2.72 times higher than that of the PI oral group (*p* < 0.05). Similarly, the *T*_max_ of the PDA‐CV@PI group was 2.01 times higher than that of the group receiving PI intravenously (*p* < 0.05). Furthermore, the *T*_max_ of the PDA‐CV@PI group was significantly higher (21.28 times) than that of the PI oral group (*p* < 0.01). The area under the curve (AUC) of the PDA‐CV@PI group was 29204.5 ± 1562.9 mg L^−1^ h^−1^, which was 1.2 times greater than that of the PI intravenous group (*p* > 0.05) and 2.7 times greater than that of the PI oral group (*P *< 0.05). The mean residence time of the PDA‐CV@PI group (100.73 ± 2.84 h) and t1/2 (53.69 ± 15.51 h) were 1.46 and 1.74 times higher than those of the PI intravenous group (*p* > 0.05), and 2.5 and 2.85 times higher than those of the PI oral group (*p* < 0.05), respectively.

### 2.4 Drug Efficacy Test in Subcutaneous Tumor‐Bearing Mouse Model

**Figure 4:**
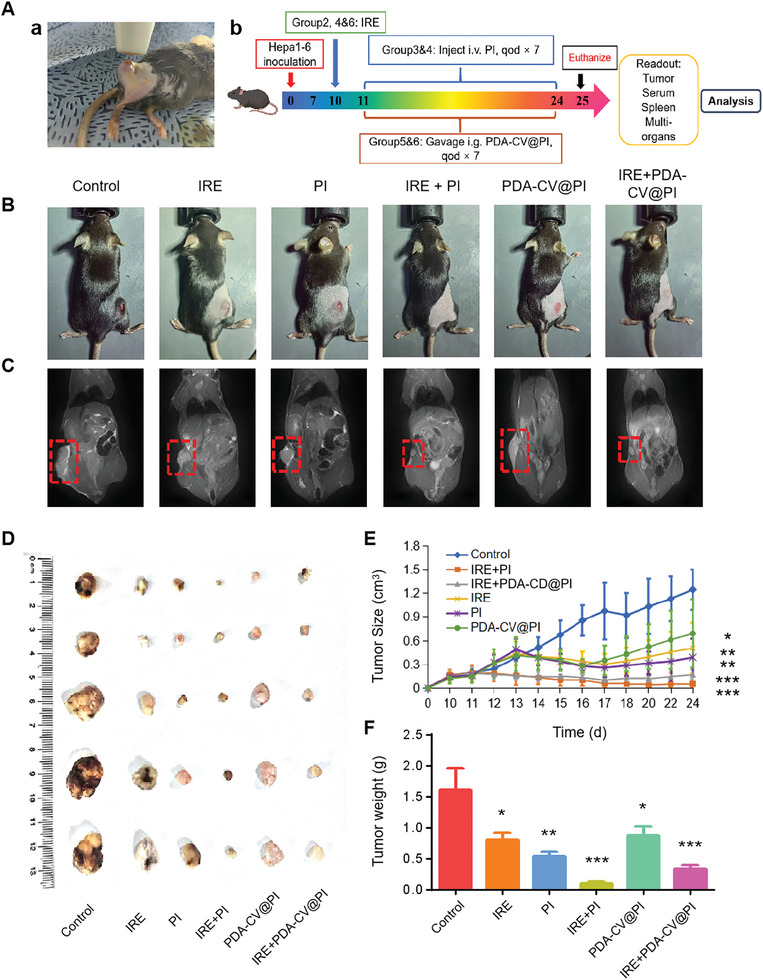
Subcutaneous tumor outcomes in mice treated with the PDA‐CV@PI combination. A) In vivo tumor inhibition by combined treatment with IRE and PDA‐CV@PI in the Hepa1‐6 tumor model. (a: Photograph of two needle electrodes inserted into Hepa1‐6 hormonal mice. b: In vivo experimental plan of IRE combination treatment); B) representative photograph of Hepa1‐6 tumor‐bearing B6‐hPD1 mice 14 days after various treatments; C) MRI T2 sequence images; D) representative photograph of dissected tumors; and E,F) tumor growth curves and tumor weight changes of B6‐hPD1 mice bearing Hepa1‐6 tumors following various treatments. Values are expressed as mean ± SD. (versus control group, *: *p* < 0.05, **: *p* < 0.01, and ***: *p* < 0.001 determined by Student's *t*‐test).

After 14 days of treatment, the tumor volumes in the mice were analyzed using a subcutaneous graft tumor model. Tumor volumes in IRE + PI and IRE + CV@PI groups were significantly decreased (*P *< 0.05 and 0.01, respectively) compared with those in the control group (**Figure** **4**A,B,D). These findings indicate that the drug‐loaded microalgae gradually released PI over time, resulting in a better anti‐tumor effect. The T2 sequence of nuclear magnetic resonance demonstrated strong signal expression in the subcutaneous tumors of all groups (Figure 4C). These tumors, located beneath the skin of the right hind leg, were elliptical in shape. The tumor volumes in the IRE + PI and IRE + PDA‐CV@PI groups were 0.0573 and 0.1780 cm^3^, respectively, compared to 1.4316 ± 0.2362 cm^3^ in the control group, 0.5303 ± 0.3573 cm^3^ in the IRE group, and 0.4261 ± 0.3573 cm^3^ in the PI group (Figure 4E,F). The IRE + PI group was injected into the tail vein, whereas the IRE + CV@PI group was administered the drug orally. Although PI can regulate T cell activity by blocking immune checkpoints, thereby promoting T cell‐mediated anti‐tumor responses, it can also cause immune‐related adverse events. The frequency of toxicity and side effects caused by PI, in order, include skin toxicity, thyroid toxicity, lung toxicity, liver toxicity, and cardiac toxicity, while gastrointestinal toxicity, other endocrine toxicities, arthritis/myositis, and other toxicities are less common.^[^ ^49^ ^]^ According to reports, 79.3% of subjects with PI experienced reactive capillary hemangiomatosis, with an increased incidence of skin capillary proliferation and significantly elevated blood transaminase levels.^[^ ^50^ ^]^ The side effects of direct PI injections are apparent. Therefore, oral administration was used in this study to reduce the side effects of PI. Direct and long‐term intravenous injections can lead to immune‐related adverse events; however, oral drug administration is relatively safe. In conclusion, compared to IRE or PI alone, the IRE + PI and IRE + PDA‐CV@PI groups displayed improved therapeutic effects, but the IRE + CV@PI group had fewer side effects. Therefore, we believe that IRE + CV@PI exhibits excellent therapeutic effects with fewer side effects.

**Figure 5:**
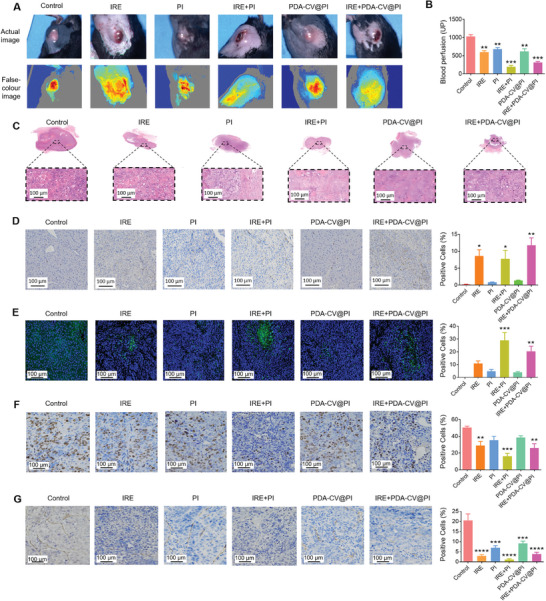
Evaluation of treatment in each group of mice. A) Distribution of subcutaneous tumor vasculature in mice; B) analysis of subcutaneous tumor vascular perfusion; C) representative photographs of pathological tumor tissues stained with H&E from each group of mice; D) immunohistochemical CD8 expression in each group 14 days post‐surgery; E) apoptotic staining conditions and their statistical results for each group; F) immunohistochemical Ki‐67 expression in each group 14 days post‐surgery; and G) immunohistochemical CD31 expression in each group 14 days post‐surgery. Values are expressed as mean ± SD. (vs control group, *: *p* < 0.05, **: *p* < 0.01, and ***: *p* < 0.001 determined by Student's *t*‐test).

The control group exhibited a greater degree of tumor indistinctness and well‐developed vascular supply, which collectively suggested a relatively high degree of malignancy. Conversely, the co‐administration group demonstrated distinct tumor boundaries and a significant reduction in tumor size compared with the control group. Meanwhile, vascular distribution in the control group was important for its abundance and expression (**Figure** **5**A,B; Figure S2, Supporting Information). However, comparing injection‐only and oral groups with the control group revealed a tendency toward reduced blood perfusion (*p* < 0.05). Moreover, the combined treatment group exhibited a notable reduction in the vascular distribution within the tumor area and blood perfusion.

Hematoxylin and eosin (H&E) staining revealed that the properties of the cancer cells in the control group closely resembled those of the initial cell lines (Figure 5C). Tumor tissues exhibit reduced cancer cell density and cellular crumpling while increasing mesenchymal connective tissue. Conversely, staining of the transplanted tumor in the IRE + PI injection and IRE + PDA‐CV@PI oral administration groups demonstrated a decrease in cancer cell density within the tumor tissues. Furthermore, most cells appeared wrinkled with increased interstitial connective tissue and mild fibrotic changes. Moreover, some cancer nests exhibited varying degrees of flaky necrosis.

Immunohistochemistry results indicated a significant increase in CD8+ cells in the combined treatment groups (Figure 5D). The IRE + PI group exhibited a significant increase in CD8+ cell expression, demonstrating that the combined use of IRE and PI can synergistically promote cytotoxic T‐cell infiltration and activation in the tumor. Furthermore, compared with the group treated with PI alone, the IRE + PDA‐CV@PI group exhibited even higher levels of CD8+ cell expression. This may be attributed to PDA‐CV, which improved the stability and bioavailability of PI, thereby achieving a superior immune activation effect in the combined treatment.

The percentage of apoptotic tumor cells was 0%, 10.93%, 4.94%, 28.97%, 4.06%, and 20.5% in the control, IRE, PI, IRE + PI, PDA‐CV@PI, and IRE + PDA‐CV@PI groups, respectively (Figure 5E; Figure S3, Supporting Information). The percentage of apoptotic cells exhibited a sharp increase in the IRE + PI and IRE + PDA‐CV@PI groups (*p* < 0.01 and 0.05, respectively).

The Ki‐67 expression rates in tumor tissues at 14 days postoperatively were 50.69%, 29.22%, 35.47%, 16.10%, 38.55%, and 25.94% in the control, IRE, PI injection, IRE + PI injection, PDA‐CV@PI oral, and IRE + PDA‐CV@PI oral groups, respectively (Figure 5F). Ki‐67 expression was significantly lower in the combined treatment groups than in the control group (*p* < 0.01 versus *p* < 0.05). The IRE + PI and IRE + PDA‐CV@PI oral groups demonstrated a significant reduction (*p* < 0.01) compared to the control group. Concurrently, CD31 expression in tumor tissues was examined using immunohistochemistry 14 days after surgery. CD31 expression levels were 20.53%, 4.29%, 6.96%, 1.09%, 9.14%, and 3.84% in the control, IRE, PI injection, IRE + PI injection, PDA‐CV@PI oral, and IRE + PDA‐CV@PI oral groups, respectively (Figure 5G). Both combined treatment groups exhibited significantly lower CD31 expression than the control group (*p* < 0.01).

### 2.5 Mechanistic Effects of PDA‐CV@PI Combined with IRE

**Figure 6:**
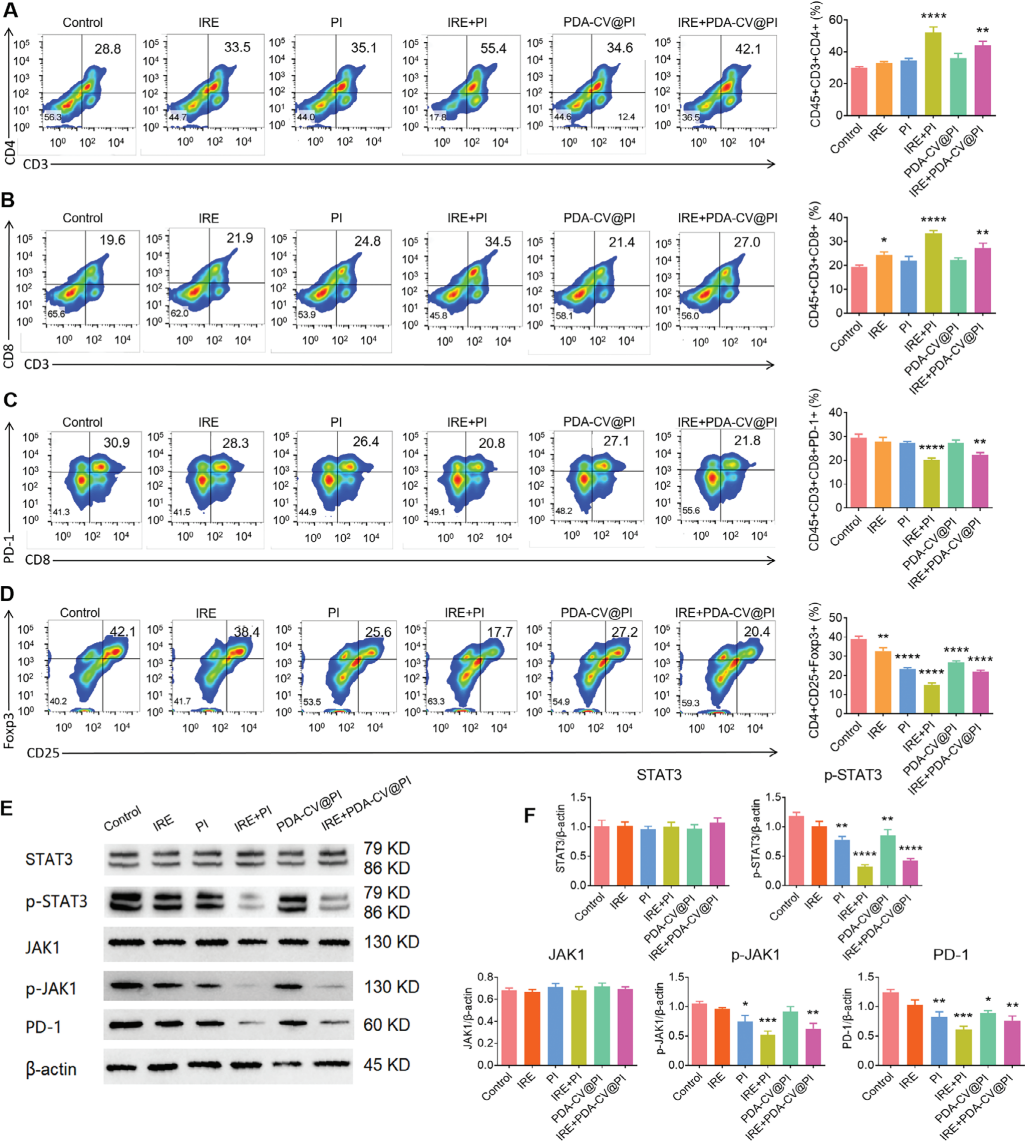
Mechanistic effects of PDA‐CV@PI combined with IRE for HCC treatment. A) CD45+CD3+CD4+; B) CD45+CD3+CD8+; C) CD45+CD3+CD8+PD‐1+; D) CD4+CD25+Foxp3+; E) WB results of PD‐1 and related pathway proteins; and F) statistical bar chart of PD‐1 and related pathway proteins by WB. Values are expressed as mean ± SD. (versus control group, *: *p* < 0.05, **: *p* < 0.01, and ***: *p* < 0.001 determined by Student's *t*‐test).

The IRE procedure was successful in all mice, with no postoperative complications. As illustrated in **Figure** **6**A–D, CD4+ and CD8+ T cells were significantly increased in the IRE + PI (52.26% ± 7.71% and 33.58% ± 2.33%) and IRE + PDA‐CV@PI (43.96% ± 6.23% and 27.42% ± 4.28%) groups 14 days after surgery, compared to the tumor control group (*P *< 0.01). Furthermore, the percentage of PD‐1 cells with CD8+ T cells was significantly lower than that in the tumor control group, with percentages of 22.46 ± 2.71% and 30.16 ± 4.14% (*P *< 0.01 and *P *< 0.05), respectively. The percentage of Tregs in both groups was significantly lower than that in the tumor control group, representing 15.18 ± 1.93% and 21.94 ± 1.69%, respectively (*P *< 0.01 and *P *< 0.05). This indicates that IRE combined with immunotherapy could enhance anti‐tumor immunity more effectively.

The levels of signal transducer and activator of transcription 3 (STAT3), phosphorylated‐STAT3 (p‐STAT3), Janus kinase 1 (JAK1), phosphor‐Janus kinase‐1 (p‐JAK1), and PD‐1 in tumor tissues were measured using Western blot (WB). The results indicated that IRE + PI and IRE + PDA‐CV@PI groups significantly suppressed the phosphorylation of JAK1 and STAT3 and the expression of PI (Figure 6E,F; Figure S4, Supporting Information). However, no significant changes were observed in JAK1 and STAT3 levels after different treatments (Figure 6E,F, Supporting Information).

### 2.6 Drug Efficacy Analysis in Orthotopic Tumor Mouse Model

**Figure 7:**
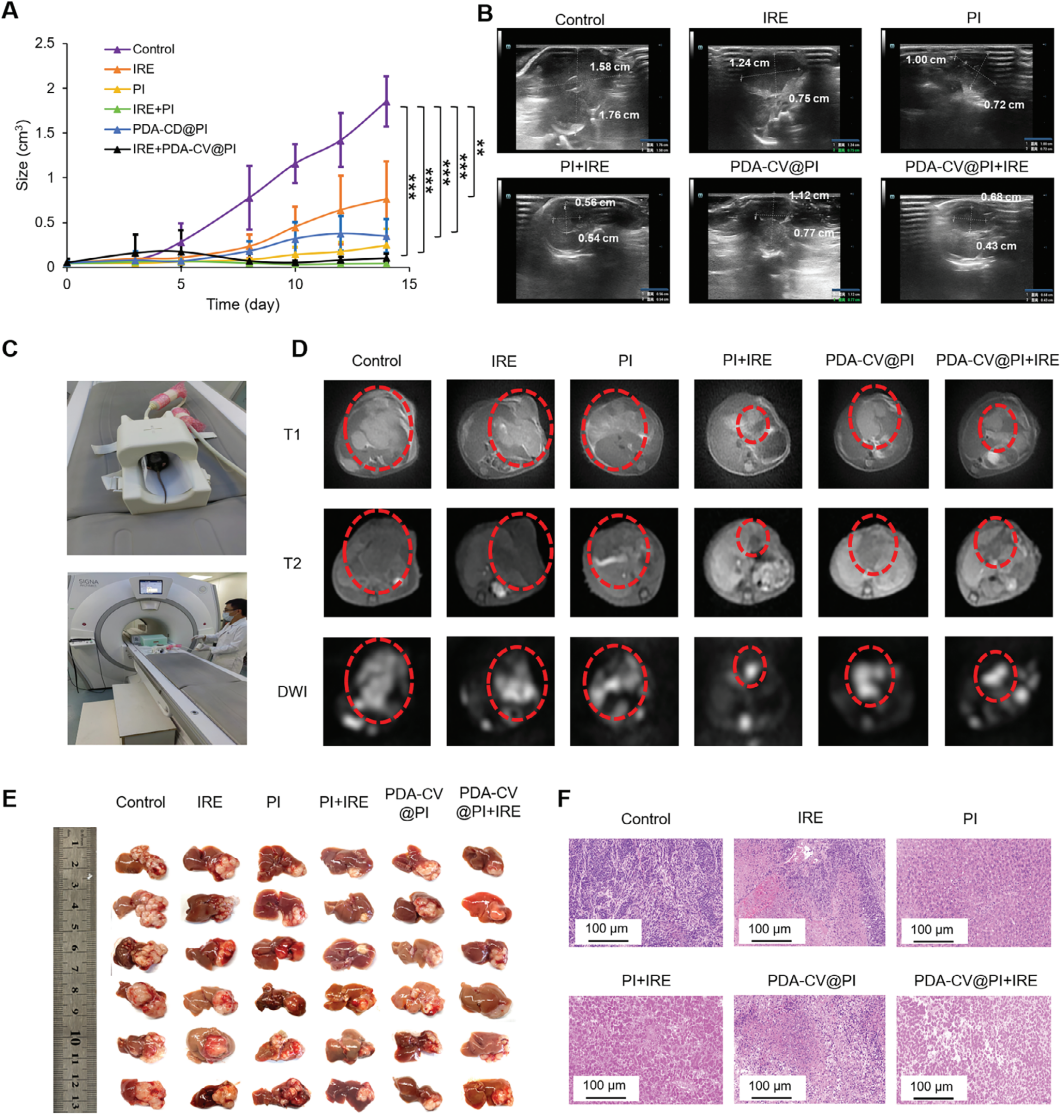
Effects of different treatments in an orthotopic HCC model. A) Tumor volume curves for each group; B) Ultrasound images of tumors; C) Images of mice during MRI examination and the tumors; D) MRI: T1, T2, and DWI sequence images; E) Representative photographs of dissected tumors; and F) Representative H&E‐stained pathological tumor tissue images from each group of mice. (versus control group, *: *p* < 0.05, **: *p* < 0.01, ***: *p* < 0.001 determined by Student's *t‐*test).

In the in vivo anti‐tumor therapy, we labeled different treatments directly, as illustrated in **Figure** **7**A. We evaluated the in vivo effects of IRE combined with CV@PI oral therapy in an orthotopic HCC model. After 14 days of treatment, no significant difference in tumor volume was observed between the groups. Over time, the tumor volume in the control group increased rapidly, whereas the IRE and PI groups demonstrated some degree of tumor suppression. However, compared with the control group and IRE or PI alone, IRE + PI injection and IRE + CV@PI treatment reduced tumor size (Figure 7B). The orthotopic tumor volume was monitored using ultrasound and magnetic resonance imaging (MRI; Figure 7C–E). After treatment, the mice were sacrificed, and the tumors were collected and photographed, revealing results consistent with the above findings (Figure 7F). H&E staining revealed the tumor cell morphology and arrangement (Figure 7G). The proliferative capacity of tumors was significantly reduced in the IRE + PI and IRE + CV@PI groups compared to that in the other groups. More apoptotic and inflammatory responses were observed in the tumor tissues of IRE + PI and IRE + PDA‐CV@PI groups, which may be attributed to the therapy‐induced immune response.

**Figure 8:**
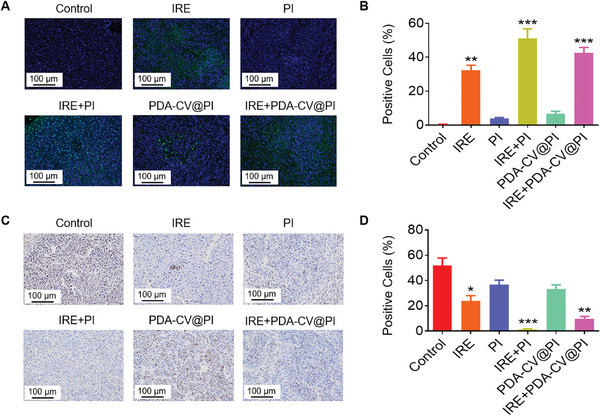
Analysis of apoptosis and immunohistochemistry induced by different treatments in an orthotopic HCC model. A) Apoptotic staining images for each group; B) statistical bar charts of apoptosis for each treatment group; C) immunohistochemical Ki67 expression in each group 14 days post‐surgery; and D) statistical bar charts of immunohistochemistry results for each group. Values are expressed as mean ± SD. (vs control group, *: *p* < 0.05, **: *p* < 0.01, and ***: *p* < 0.001 determined by Student's *t‐*test).

The IRE group exhibited a significant increase in apoptosis, whereas the PI injection alone and oral CV@PI alone groups did not differ significantly from the control group (**Figure** **8**A,B; Figure S5, Supporting Information). The IRE + PI and IRE + CV@PI groups demonstrated a significant increase in the number of apoptotic cells compared with the control group. Immunohistochemical analysis of the tumor tissue 14 days after surgery revealed a similar trend, with the IRE group indicating a significant increase in apoptosis (*p* < 0.05), whereas the PI injection alone and oral CV@PI alone groups did not differ significantly from the control group (Figure 8C,D). The IRE + PI and IRE + CV@PI groups demonstrated a significant increase in the apoptosis index compared to the control group (*p* < 0.005 and *p* < 0.01, respectively).

**Figure 9:**
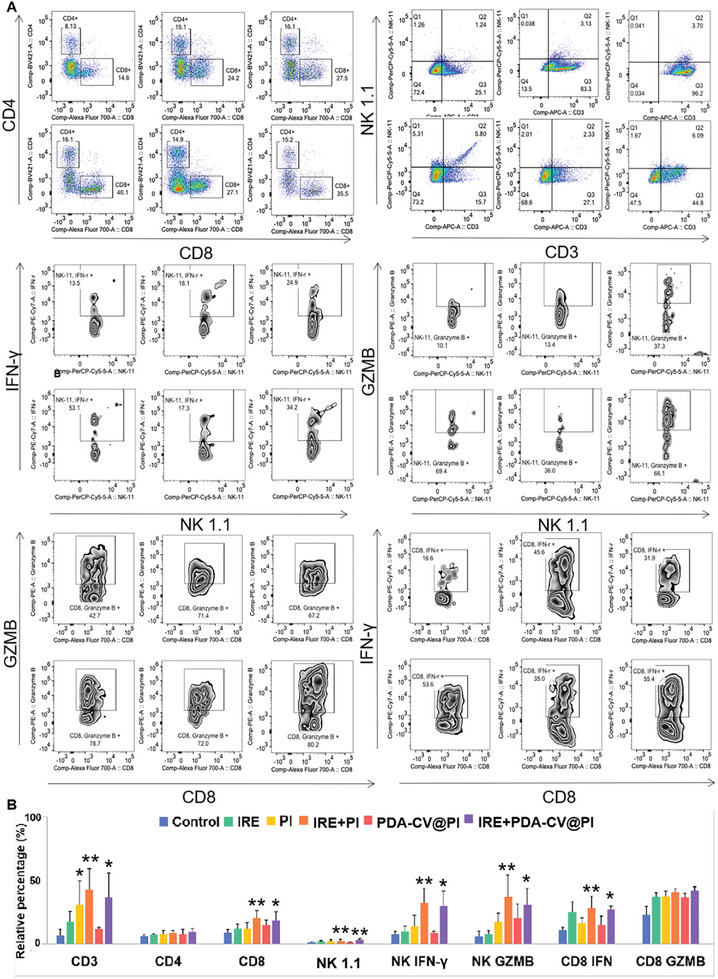
Mechanistic effects of PDA‐CV@PI combined with IRE for HCC treatment, as indicated by flow cytometry. A) Flow cytometry results of tumor tissue from each treatment group (CD3, CD4, CD8, NK1.1, NK IFN‐γ, NK GZMB, CD8 IFN, and CD8 GZMB); and B) statistical graphs of flow cytometry results for each treatment group. Values are expressed as mean ± SD. (vs control group, *: *p* < 0.05 and **: *p* < 0.01, determined using the Student's *t*‐test).

All the mice underwent a successful IRE procedure, and no postoperative complications were observed. We comprehensively analyzed immune cell surface markers in the tumor microenvironment after different treatments using flow cytometry. Particular attention was paid to the expression levels of key markers, including CD3, CD4, CD8, natural killer (NK‐1.1), interferon‐gamma (IFN‐γ), granzyme B (GZMB), CD8 IFN, and CD8 GZMB. The IRE group demonstrated significantly increased expression of CD8 and NK‐1.1 compared to the control group (**Figure** **9**A,B). Moreover, PI treatment increased the expression of CD3, CD8, and NK‐1.1, which may indicate that PI enhances immune cell function and tumor antigen presentation ability by relieving T cell immune suppression. The IRE + PI injection and IRE + CV@PI oral groups revealed a significant increase in the expression of multiple immune cell surface markers, particularly CD8, NK‐1.1, and NK GZMB, compared to the other groups. These results indicate that the combined therapy may enhance the anti‐tumor immune response by activating various immune cells, including T and NK cells.

### 2.7 Biosafety Evaluation

**Figure 10:**
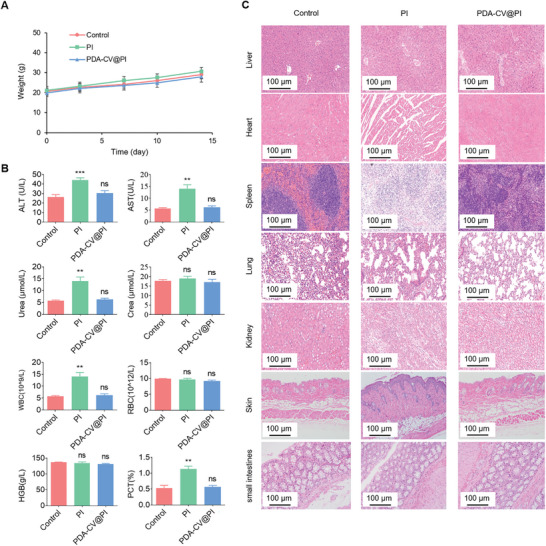
Preliminary toxicity analysis of PDA‐CV@PI. A) Body weight changes in mice in different treatment groups; B) biochemical and routine blood tests; and C) H&E staining images of the liver, heart, spleen, lungs, kidneys, small intestine, and skin of mice 14 days after surgery (normal, PI injection, and PDA‐CV@PI oral groups. Values are expressed as the mean ± SD. (vs the control group, ns: *p* > 0.05, *: *p* < 0.05, **: *p* < 0.01, and ***: *p* < 0.001 determined by Student's *t*‐test).

Biosafety is crucial for the clinical application of pharmaceutical formulations. First, we conducted blood biochemical tests 14 days after treatment. There was no significant difference in dynamic changes in body weight among the three groups of mice (**Figure** **10**A). Compared with the control group, the levels of aspartate transaminase (AST) and alanine transaminase (ALT) in the PI group were significantly elevated (*p* < 0.01). Conversely, no significant differences were observed in any of the indicators between PDA‐CV@PI and control groups (*p* > 0.05). The above results indicate that PI caused liver function impairment, whereas PDA‐CV@PI was relatively safe. Second, to further demonstrate that the oral formulation of the immune checkpoint inhibitor provides a less toxic alternative, we examined routine blood indicators, including white blood cell count (WBC), red blood cell (RBC), hemoglobin (HGB), and procalcitonin (PCT) (Figure 10B, Table S3, Supporting Information). WBC and PCT levels in the PI group were significantly elevated compared to those in the control group (*p* < 0.01). However, no significant differences were observed in any of the indicators between PDA‐CV@PI and control groups (*p* > 0.05). These results suggest that the PDA‐CV@PI group is safer than the PI group. Finally, the hearts, livers, spleens, lungs, kidneys, small intestines, and skin were collected from the mice in the three groups for H&E staining. The results revealed that compared with the control group, the skin tissue in the PI group exhibited mild hyperplasia and thickening of the squamous epithelium, with parakeratosis visible on the surface. Numerous inflammatory cells infiltrated the dermis along with a few eosinophils. The sebaceous glands and hair follicle structures were also observed. No clear loose edema was observed. Fat and muscle tissues were visible in the subcutaneous layer, accompanied by scattered inflammatory cell infiltration. However, no significant damage was observed in any of the tissues in the PDA‐CV@PI group (Figure 10C). These results indicate that PDA‐CV@PI exhibits excellent biosafety.

### 2.8 Gut Microbiota and Metabolic Pathway Analysis

**Figure 11:**
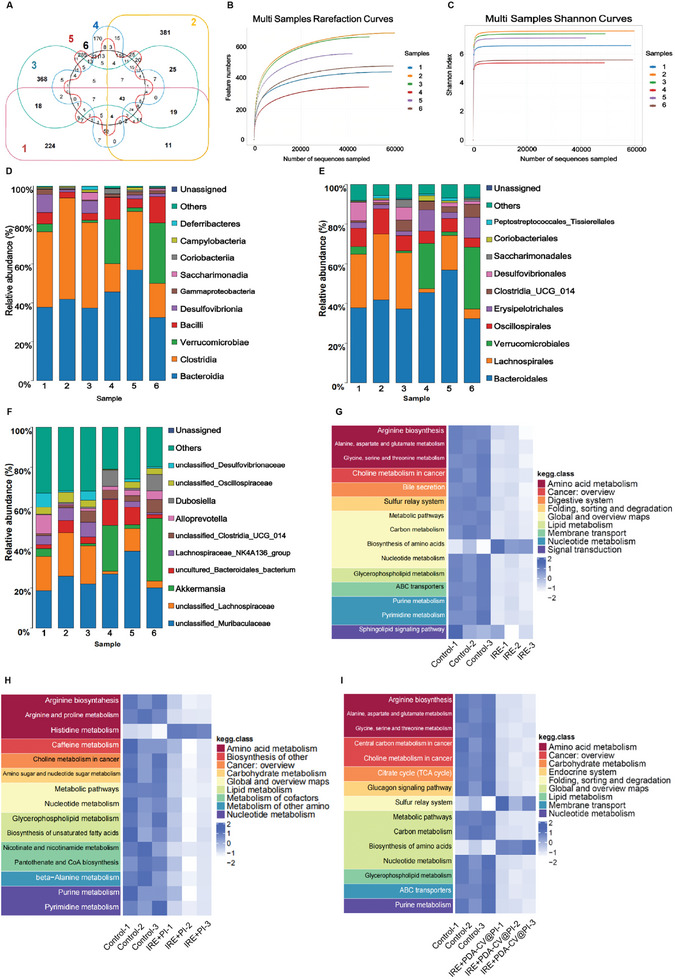
Intestinal microbiota and metabolomic analysis results for different treatment groups. A) Venn diagram of differentially expressed genes among the treatment groups; B) multisample rarefaction curves for sequencing samples from each group; C) Shannon curves for sequencing samples from each group; D–F) intestinal microbiota analysis diagrams for each treatment group; G) Kyoto Encyclopedia of Genes and Genomes (KEGG) pathway enrichment results for differentially expressed genes in the IRE group alone; H) KEGG pathway enrichment results for differentially expressed genes in the IRE + PI group; and I) KEGG pathway enrichment results for differentially expressed genes in the IRE + PDA‐CV@PI group.

Venn diagrams of the classified operational taxonomic units of the intestinal flora of the mice in each group are illustrated in **Figure** **11**A. These diagrams demonstrate that the intestinal flora composition in the control group was more similar to that in the treatment group, whereas the combined treatment group exhibited the highest similarity in intestinal flora composition. The samples were sequenced in sufficient quantities, and the sequencing depth was sufficient to cover most of the microbial colonies for data analysis (Figure 11B). The α‐diversity analysis illustrated the diversity and abundance of flora species (Figure 11C). The Shannon index plot indicated a significant increase in the intestinal flora abundance in IRE + PI and PDA‐CV@PI oral groups compared with that in the control group (*p* < 0.01) and a significant decrease in IRE + PI and IRE + PDA‐CV@PI groups (*p* < 0.05). However, the combined treatment groups exhibited no significant differences in the Shannon index.

Figure 11D illustrates the distribution of intestinal flora at each level. Four flora species, *Bacteroidetes*, *Firmicutes*, *Verrucomicrobiota*, and *Desulfobacterota*, were predominant at the class level. The relative abundance of *Desulfobacterota* in the intestinal flora of mice in the combined treatment groups (d and f) was reduced compared to that in the control group, and the *Firmicutes*/*Bacteroidetes* ratio (F/B value) decreased. Conversely, the relative abundance of *Verrucomicrobiota* was significantly increased, indicating an improvement in the intestinal flora composition of the mice. *Bacteroidales*, *Lachnospirales, Verrucomicrobiales*, *Oscillospirales*, and *Erysipelotrichales* were predominant at the family level (Figure 11E). Similarly, compared to the control group, the relative abundances of *Bacteroidales*, *Erysipelotrichales*, *Verrucomicrobiales*, and *Clostridia*_UCG_014 increased in the intestinal flora of mice in the combination treatment group, whereas those of *Lachnospirales*, *Desulfovibrionales*, and *Oscillospirales* decreased. However, no significant difference was observed in the flora between the two groups compared with that in the combined treatment groups (*P *> 0.05). Figure 11F illustrates the distribution of the intestinal flora of the mice at the genus level. Compared to the control group, the relative abundance of unclassified*_Muribaculaceae, Akkermansia*, *uncultured_Bacteroidales_bacterium, Dubosiella*, and unclassified_*Clostridia_ UCG_014* was increased in the intestinal flora of mice in the combined treatment group. Meanwhile, the relative abundance of unclassified*_Lachnospiraceae, Alloprevotella*, and *unclassified_Desulfovibrionaceae* was decreased. However, no significant difference was observed in the intestinal flora between the two groups when the combined treatment groups were compared (*P *> 0.05).

Subsequently, metabolic pathway analysis was performed. The IRE group displayed upregulated amino acid biosynthesis compared to the control group (Figure 11G) but downregulated arginine biosynthesis, alanine, aspartate, glutamate, glycine, serine, and threonine metabolism, and choline metabolism in cancer. Similarly, the IRE + PI injection group exhibited upregulated lysine degradation and downregulated arginine biosynthesis and alanine, aspartate, glutamate, glycine, serine, and threonine metabolism compared with the control group (Figure 11H). Moreover, the PDA‐CV@PI oral group demonstrated an upregulated sulfur relay system and amino acid biosynthesis, along with downregulated arginine biosynthesis, alanine, aspartate, glutamate, glycine, serine, threonine, central carbon metabolism, and choline metabolism in cancer compared to the control group (Figure 11I).

## 3 Discussion

Currently, IRE is a viable option for the initial management of small HCC and serves as a non‐thermal method for localized oncology.^[^ ^51^ ^]^ Compared to thermal ablation, IRE is highly resistant to the “heat‐sink effect.” It protects against damage to adjacent heat‐sensitive structures such as the bile ducts, gallbladder, and hepatic peritoneum. Furthermore, IRE seldom affects the extracellular matrix, thereby maintaining the regeneration and healing ability of the liver.^[^ ^52^ ^]^ The induction of apoptosis, which can regulate immune tolerance, is the primary approach for treating tumors. The prevailing evidence suggests that the combination of immunotherapy and IRE presents a challenge in achieving synergistic effects. Numerous studies have demonstrated that IRE is more effective than thermal or cryoablation in stimulating T‐cell immunity.^[^ ^53^ ^]^ For instance, a previous study reported significantly improved survival rates in mice treated with a combination of IRE and a PD‐1 antibody in the control of orthotopic tumors compared to that of mice treated with PD‐1 or IRE alone.^[^ ^54^ ^]^ CD8+ *T*‐lymphocyte infiltration occurred approximately seven days after treatment in IRE‐treated tumor tissues,^[^ ^55^ ^]^ and *T*‐lymphocyte infiltration occurred one day after IRE treatment.^[^ ^56^ ^]^ These reports indicate a potential synergistic effect of IRE combined with immunotherapy.

Recent studies have summarized the factors contributing to the improved immunotherapeutic outcome of IRE in combination with PD‐1 in mouse tumor models, with three potential immunological mechanisms that may be responsible for enhancing tumor immune responses and collaborating to achieve anti‐tumor effects. IRE boosts tumor immunogenicity by generating neoantigens from destroyed tumor cells, stimulating an anti‐tumor immune response. Furthermore, it enhances the infiltration of specific T cells by promoting the synthesis and secretion of molecular patterns linked to tumor cell destruction.^[^ ^57^ ^]^ Antigen‐presenting cells can induce specific immune responses when antigens are absorbed in the periphery of the cell. Furthermore, PD‐1 antibodies can increase the stimulation of tumor antigen‐targeting CD4+ and CD8+ T cells following IRE, thereby enhancing the anti‐tumor effect of the immune system.^[^ ^58^, ^59^ ^]^ Previous research has revealed that IRE can alter the immunosuppressive tumor microenvironment, shifting it from anti‐inflammatory to pro‐inflammatory, thereby mediating acute inflammatory responses. This is primarily due to the pro‐inflammatory and pro‐immune responses of IRE.^[^ ^58^ ^]^ IRE simultaneously induces efflux and influx of potassium and other ions by creating holes in the cell membrane. Potassium ion outflow induces scorch death.

Tumor cells can converge CD8+ T lymphocytes inside the tumor, even if a small amount of scorch death occurs after treatment, thus effectively inhibiting tumor metastasis.^[^ ^60^ ^]^ The reversal of immunosuppression by eliminating Treg cells revealed the anti‐immunosuppressive effects of IRE, providing an opportunity for combination immunotherapy to maximize the therapeutic efficacy of PI.^[^ ^61^ ^]^ Furthermore, intra‐tumoral hypoxia is a key regulator of immunosuppression, and IRE can improve intra‐tumoral hypoxia, thereby enhancing anti‐tumor immunity.^[^ ^62^ ^]^ However, studies have demonstrated that solitary IRE therapy often leads to local recurrence and disease progression after surgical intervention.^[^ ^63^ ^]^ This suggests that IRE can enhance the immune response against tumors by directly eliminating cancer cells and indirectly stimulating the T‐cell immune response. However, this is insufficient to prevent tumor recurrence and progression. Although this immune enhancement may be insufficient to prevent tumor recurrence, it provides the opportunity to combine IRE with immunotherapy.

Tumors promote their growth by altering the mechanisms that suppress the immune system and generate immune evasion. The PD‐l/PD‐L1 immune checkpoint plays a vital role in tumor progression. Monoclonal antibodies disrupt the interactions between PD‐1 and PD‐L1, increase T cell toxicity, restore the immune system's ability to monitor and eliminate tumors, and inhibit CD8+ T cell transformation to Treg cells.^[^ ^64^ ^]^ Recent research has established a correlation between the efficacy of PI and the presence of CD8+ cells that have infiltrated the tumor.^[^ ^65^ ^]^ Treg cells, a subset of CD4+ T cells that express Foxp3, have been demonstrated to suppress the ability of the immune system to produce an immune response against tumors and promote tumor immune evasion. The effectiveness of numerous immunosuppressive drugs is linked to their capacity to directly or indirectly inhibit Tregs. Immune checkpoint inhibitors targeting PD‐1/PD‐L1 have displayed favorable efficacy in clinical settings.^[^ ^66^ ^]^ However, the effectiveness of immunotherapy for HCC is limited, with a response rate of <30%, which could be attributed to immune microenvironment tolerance, inadequate tumor antigen exposure, and insufficient infiltration of T cells in HCC.^[^ ^67^, ^68^ ^]^ Therefore, immunotherapy is commonly used as an adjunctive therapy, along with other traditional therapies, to improve the overall treatment outcomes, potentially hindering tumor reappearance and dissemination.

Conversely, electroporation induces apoptosis and necrosis by disrupting the integrity of the tumor cells and lysosomal membranes. This process also produces numerous highly antigenic cell fragments, effectively eliminating tumor cells and increasing tumor antigen exposure. This creates a favorable environment for the effectiveness of PD‐1 antibodies and prevents immune evasion by tumor cells. Consequently, it is imperative to recognize the advantages of combining immunotherapy and physical ablation, as they improve the immune response against tumors and reduce the probability of recurrence after ablation.

Despite the effectiveness and significant therapeutic potential of PI, patients often discontinue treatment owing to the high cost, inconvenience, and discomfort associated with it, as well as their inability to endure prolonged injectable administration. Quality of survival is significantly affected by these factors. Conversely, oral administration has notable advantages, including high compliance, self‐administration, cost‐effectiveness, and avoidance of damage to the local skin or mucous membrane, making it suitable for long‐term patient administration. However, the advancement of oral delivery systems for monoclonal antibodies is significantly impeded by biochemical conditions within the gastrointestinal tract, hepatic excretion, limited control over drug release duration, and poor bioavailability, which collectively present substantial barriers. Oral drug delivery remains the preferred method owing to its multiple benefits. There is increasing interest in the development of advanced carriers for the oral delivery of different therapeutic agents. Because of various obstacles to absorption in the digestive system, conventional methods of oral drug administration have drawbacks, such as liver elimination, limited effectiveness in specific areas, low oral absorption rates, and vulnerability to negative consequences. The “composite drug carrier” is a novel type of carrier that combines the advantages of both “biological carriers” and “synthetic carriers,” thereby overcoming the disadvantages of traditional oral drug delivery systems. It can improve medicinal efficacy, oral bioavailability, and patient compliance.

Peptide delivery using plant cells as carriers has recently become increasingly popular.^[^ ^69^ ^]^ This delivery vehicle offers various advantages, including uniform size, low cost, easy scale‐up, excellent biosynthetic and modification capabilities, efficient encapsulation, and protection of biopharmaceutical proteins from degradation in the gastrointestinal system, thereby improving oral drug bioavailability.^[^ ^70^ ^]^ Currently, this group has researched and developed several microalgal delivery systems. Microalgae are among the most prevalent naturally occurring biological materials and are abundant in freshwater, marine, and terrestrial habitats. They are rich in protein, dietary fiber, polysaccharides, vitamins, and minerals and are produced and developed in large quantities as health foods, functional foods, and dietary supplements.^[^ ^71^ ^]^ It demonstrates potential as an antioxidant, anti‐inflammatory, anti‐tumor, anti‐bacterial, anti‐viral, and anti‐allergic substance, playing a crucial role in the biomedical and pharmaceutical sectors.^[^ ^72^ ^]^

Furthermore, microalgae have inherent water channels within their cell membranes that facilitate the exchange of extracellular and intracellular materials by enabling the passage of specific ions and molecules between the internal and external environments of living cells.^[^ ^73^, ^74^ ^]^ Because of its large size, the biomolecule FITC‐BSA can be loaded into microalgal cells through connecting pores.^[^ ^75^ ^]^ Microalgal cells are well‐suited for drug transportation because of the presence of these natural channels, as they can absorb specific drug molecules through osmotic pressure. Additionally, the presence of hydroxyproline‐rich glycoproteins in the external layer of microalgae contributes to their high electronegativity. These microorganisms can attract positively charged substances or magnetic nanomaterials via electrostatic interactions.^[^ ^76^, ^77^ ^]^ Moreover, specific growth factors present in microalgae enhance the immune system.^[^ ^78^ ^]^

Additionally, it contains an “acidic polysaccharide” that triggers the production of interferon, thereby improving the immune response and augmenting its ability to combat diseases.^[^ ^79^ ^]^ Biomedical applications are among the most important and extensively researched areas for PDA applications. Extensive research has been conducted on PDA in the medical domain because of its remarkable surface adhesion to organic or inorganic substrates,^[^ ^80^ ^]^ acid resistance,^[^ ^81^ ^]^ and favorable biocompatibility.^[^ ^82^ ^]^ These properties enable drug‐loaded CV to withstand gastric acid and to adhere waterproof to the small intestine.

Therefore, this study combined immunotherapy and IRE using CV (a negatively charged surface) as a carrier for medication. An electrostatic precipitation technique was used to load and transport the PI, which was then enclosed in PDA as a protective covering. PDA‐coated CV@PI was successfully prepared to increase the intestinal retention time and bioavailability of the drug, ultimately leading to improved efficacy. CV demonstrated a significant drug‐loading capacity and consistent particle size in this oral system, enabling efficient protection of the drug from degradation in the acidic environment of the stomach. The drug delivery system can remain in the intestinal tissues for an extended duration because of the structure of the PDA shell, which can be easily absorbed by the villi in the intestine, thereby accomplishing secure and gradual release of PI. Moreover, orally administered PDA‐coated CV@PI exhibited an excellent biosafety profile as it did not induce adverse reactions.

This study collected data from different groups at 14 days postoperatively in an in vivo trial. The combination of IRE + PI and oral administration of CV@PI significantly enhanced T‐cell infiltration in tumor tissues compared to that in the control group 14 days after surgery, thereby compensating for the inadequate effectiveness of PI. Furthermore, combining the two therapies resulted in a significant increase in the infiltration of CD4+ and CD8+ cells within tumor tissues and a notable decrease in Treg cells, resulting in a significant reduction in tumor size or even complete eradication in mice. These findings indicate that combining IRE and PI results in a more significant immune response against tumors and a higher impact on reducing tumor burden. Concurrently, we revealed that IRE hindered JAK1 and STAT3 phosphorylation 14 days after surgery, consequently suppressing PD‐L1 expression. This phenomenon could be attributed to the release of multiple tumor antigens, which stimulate the inflammatory reaction and initiate the immune system, thereby activating the autoimmune response. These findings indicate that combining IRE with a PI injection and IRE with oral administration of a PI derived from microalgae can successfully hinder the initiation of the immunosuppressive mechanism and control the transformation of CD4+ and CD8+ T cells into Tregs. Therefore, there was an increase in the number of CD4+ and CD8+ T cells and a decrease in the number of Tregs. IRE intervention compensated for PI insufficiency, ultimately boosting the immune response and suppressing tumor progression. Furthermore, the IRE + PDA‐CV@PI oral group effectively suppressed tumor growth and improved the anti‐tumor immune response, similar to the IRE + PI group. Notably, these positive outcomes were achieved without adverse effects, thereby offering a promising avenue for liver cancer immunotherapy.

The human intestinal tract hosts a population of ≈10^14^ bacteria that regulate the digestion and absorption of the ingested diet and are important in determining the energy conversion rate. The results of this study demonstrated that the combined treatment significantly increased intestinal flora diversity and abundance in colon cancer mice, and activated immune function by regulating lipid metabolism in the intestinal flora. *Bacteroidetes, Firmicutes, Actinobacteria*, and *Proteobacteria* comprised 98% of the intestinal flora at the portal level. The intestine and liver are directly linked through the portal vein, influencing hepatic lipid metabolism through the “gut–liver axis”.^[^ ^83^ ^]^ These findings confirmed that the tumor control group increased the F/B value in the intestinal flora of mice but decreased it in the combined treatment group, implying that the combined treatment group could regulate and improve the disorders of intestinal flora in mice. Analysis of changes in intestinal flora composition revealed that the combined treatment significantly increased the abundance of *Akkermansia*. It has been found that *Akkermansia* can assist various anti‐tumor drugs in inhibiting tumor growth, thereby improving the objective response rate and prolonging the patients' overall survival,^[^ ^84^ ^]^ particularly with the focus of recent research on immune checkpoint inhibitors demonstrating a synergistic effect.^[^ ^85^ ^]^ Consequently, increasing the abundance of intestinal Akkermansia is a novel strategy for adjuvant HCC drug therapy.

Various initial validations have substantiated the systemic immune alterations induced by physical ablation techniques in HCC. Additional comparative research is required to evaluate variations in the strength of immune inflammation caused by ablation and the characteristics of immunomodulatory effects among different ablation techniques. Furthermore, it is imperative to investigate an optimal ablation method that can be effectively combined with immunotherapy in patients with liver cancer. Most studies have demonstrated the therapeutic advantages of combining ablation and immunotherapy compared with individual modalities. Future explorations of combination strategies should focus on two directions.

1. For patients with early‐stage liver cancer, immunotherapy can contribute to the complete eradication of the lesion through ablation and further prevent residual tumor recurrence.
2. Ablation can be used as a component of combination therapy with immunotherapy for intermediate‐to‐advanced liver cancer because it can decrease the tumor burden, induce alterations in the tumor microenvironment, and improve immunotherapy efficacy. There remains a need to determine the optimal timing for physical ablation and immunotherapy, ranging from extensive human trials to animal studies, to develop a combined strategy to maximize the translation into a positive prognosis.

## 4 Conclusion

We successfully developed a PDA‐CV@PI, a novel oral composite system that combines the benefits of a “biological carrier” and a “synthetic carrier.” This system effectively protects monoclonal antibodies from degradation in the gastric acid environment and facilitates their absorption by the intestinal villi. This method enables prolonged retention of the drug delivery system in intestinal tissues. Consequently, it optimizes the oral bioavailability of monoclonal antibodies by facilitating their safe and gradual release. The carrier has good biodegradability as it gradually degrades in the intestinal tract and is excreted from the body after drug release. Furthermore, considering the possible combined benefits of IRE and microalgae, our use of IRE + PDA‐CV@PI proved to be a successful approach to enhance the overall anti‐cancer immune response and promote the development of lasting immune memory following complete tumor elimination. Using readily available natural materials, we successfully developed a novel strategy combining oncological physiotherapy and immune antibody CV for oral application, demonstrating a high translational potential in cancer physiotherapy combination therapy.

## 5 Experimental Section

### Resources

PI, Camrelizumab, was obtained from Jiangsu Hengrui Pharmaceutical Co., Ltd., China. The CV and BG11 media were procured from Guangyu Biotechnology (Shanghai, China). CV was cultured in BG11 medium at 25 °C for 24 h under light. After culturing, CV was collected by centrifugation at 3000 × *g* for 10 min and washed three times with deionized water after collection. Dopamine (purity > 98%) was procured from Shanghai Yuan Ye Biotechnology Co. Ltd. Trypsin‐ethylenediaminetetraacetic acid (EDTA), Dulbecco's modified Eagle medium (DMEM), and fetal bovine serum (FBS) were obtained from Gibco BRL (Burlington, Ontario, Canada). All the remaining chemicals were of the utmost quality that could be obtained from the market.

### Preparation and Characterization of PDA‐CV@PI

CV was cultivated in BG11 medium supplemented with 0.1% ferric ammonium green citrate, 0.1% citric acid, 0.1% CaCl_2_‐_2_H_2_O, 0.1% EDTANa_2_, 0.1% K_2_HPO_4_, 0.1% MgSO_4_‐_7_H_2_O, Na_2_CO_3_, 0.2% NaCl, and 0.1% trace metal solution. Cultivation was performed by shaking at 250 rpm and 25 °C. The PI solution (1000 g mL^−1^) was combined with the CV suspension (500 g mL^−1^) and stirred at room temperature for 1 h. After centrifugation for 10 min at 3000 × *g*, the electrostatically adsorbed PI‐saturated CV was collected. The collected supernatant was washed thrice with deionized water before use in subsequent experiments. The dopamine hydrochloride solution was aspirated, combined with 10 mL of Tris buffer (50 mm, pH 8.5), and stirred using a magnetic stirrer at 25 °C for 8 h. Subsequently, the liquid was separated by spinning at 12 000 rpm for 10 min, washed three times with deionized water, diluted with a suitable quantity of deionized water, and stored. Subsequently, the PDA solution (pH 5.2) was supplemented with 5 mg CV@PI and stirred at 300 rpm for 12 h at 4 °C. The suspension was then centrifuged at 13 000 rpm for 5 min at 4 °C. The resulting sediment was collected and rinsed thrice with deionized water before being stored in a refrigerator at 4 °C.

The PI adsorption rate in the supernatant was measured at 280 nm using a UV‐2600 spectrophotometer (Shimadzu, Kyoto, Japan). This analysis was performed by comparing the results with those of the standard curve of the PI solution. The average particle size and zeta potential were measured using an LS‐909 Laser Particle Sizer (Malvern Panalytical Company, Guangdong, China). Bright‐field and fluorescence images were obtained using an optical microscope (Zeiss, Oberkochen, Germany). After fixing and dehydrating the samples, CV, CV@PI, and PDA‐CV@PI images were captured using a scanning electron microscope (SU‐8010, Hitachi, Japan).

### In Vitro Cumulative Release Study

The samples were suspended in 2 mL of release medium at different pH levels (1.2, 6.8, and 7.4). The mixture was stirred continuously at 80 rpm at 37 °C. The liquid was collected at specific sampling intervals (5, 10, 20, 30, and 45 min; 1, 2, 4, 5, 6, 8, 10, 12, 14, 22, and 24 h) by centrifugation at 3000 × *g* for 10 min. Subsequently, equal volumes of the same or different release media were used to replace the collected liquid. The supernatants of PI, CV@PI, and PDA‐CV@PI were analyzed using UV spectrophotometry. The cumulative release rate at each time point was determined using the following equation: [cumulative release rate = quantity of PI absorbed in CV/quantity of PI in the PI sample (including both unadsorbed and adsorbed total PI). Subsequently, the cumulative release curves were plotted.

### Evaluation of Cellular Toxicity of PDA Encapsulated CV–In Vitro Cytotoxicity Assay

Hepa1‐6 cells were selected, and a control group with normal conditions and a group with PDA‐CV@PI (0.3, 1, and 3 mg) were established. The concentration of Hepa1‐6 cells was reduced to 5 × 10^4^ cells mL^−1^. Subsequently, 90 µL of the cell suspension was added to each well of a 96‐well sterile plate and incubated for 12 h. For the microalgae group, 100 µL of PDA‐encapsulated CV@PI (0.3, 1, and 3 mg) was added to each well, and 100 µL of complete cell culture medium was added to the control group. The cells were examined under a microscope after 24, 48, and 72 h. Afterward, 100 µL of 10% cell counting kit‐8 solution was added to each well and incubated for 2 h. The absorbance was measured at 450 nm to determine relative cell viability using the following formula:

(1) $$\mathrm{The}\;\mathrm{cell}\;\mathrm{viability}\left(\%\right)=\frac{\left(X_{1}-X_{0}\right)}{\left(X_{2}-X_{0}\right)}\times 100\%$$

Blank control wells were used for zero calibration, and the formula was used to calculate the relative activity rate of each culture well, where *X*_1_ denotes the absorbance of the experimental sample group, *X*_2_ represents the absorbance of the normal control group, and *X*_0_ indicates the absorbance of the blank control group.

### Quantification of Cellular Reactive Oxygen Species (ROS)

In a 6‐well plate, 5 × 10^5^ Hepa1‐6 cells were seeded into each well. After grouping and 48 h of experimental treatment, the cells were thoroughly rinsed three times with high‐glucose DMEM. Each well was then supplemented with 1 mL of 10 µmol L^−1^ DCFH‐DA solution and incubated for 30 min in a cell culture incubator. The fluorescence intensity of cells in each group was analyzed using a flow cytometer (BD Biosciences, San Jose, CA). Data were analyzed using FlowJo software (version 10.8).

### Dual Staining of Live/Dead Cell

In a 6‐well plate, 1 × 10^5^ Hepa1‐6 cells were seeded into each well. After 48 h of experimental grouping, the cells were rinsed thrice with phosphate‐buffered saline (PBS) and subsequently exposed to PBS containing calcein‐AM at 37 °C for 30 min. Staining was performed for 5 min by adding 100 µg mL^−1^ propidium iodide solution. The cells were then rinsed once with PBS. Finally, 100 µL of PBS was added to facilitate the observation of cell staining and the acquisition of photographic evidence using a fluorescence microscope.

### In Vivo Biodistribution of Drugs Administered by Gavage

Twelve mice were selected and subjected to fasting. The mice were weighed and randomly divided into two groups, with six mice per group. One group was administered PDA@FITC‐CV by gavage, whereas the other group was administered FITC‐CV. The time of administration was recorded. The FITC‐CV group was used as the control group. After gavage, the mice were euthanized at 0.5, 1, 3, 6, 24, and 48 h. The gastrointestinal tract and other organs, including the heart, spleen, liver, kidneys, and lungs, were extracted from the mice. Fluorescence imaging was conducted using the Small Animal In Vivo Imaging System, with the following parameters for fluorescence imaging: FITC excitation and emission wavelengths of 460–550 nm and 520–530 nm, respectively. The exposure time was 1 s.

### In Vivo Pharmacokinetic Studies

The blood concentration of SHR‐1210 was measured to analyze the pharmacokinetic (PK) parameters of each drug, including half‐life (t1/2), time to reach peak concentration (*T*_max_), peak concentration (*C*_max_), and area covered by the concentration–time curve (AUC). In the first cycle, plasma samples were collected for PK curve analysis of SHR‐1210 at various time intervals: 5 min (±2 min), 0.5 h (±5 min), 2 h (±5 min), 6 h (±5 min), 24 h (±3 min), 48 h (±5 min), 72 h (±6 min), 96 h (±6 min), and 144 h (±5 min) before drug administration. The specimens were stored at −80 °C until analysis. Serum levels of SHR‐1210 were measured using an enzyme‐linked immunosorbent assay. A standard intraocular blood sample was administered to all six mice, and blood was collected in a 1.5 mL centrifuge tube coated with sodium heparin. After 15 min of centrifugation at 3500 rpm, the supernatant was collected and stored at −80 °C. The PD‐1 stock solution had a concentration of 800 µg mL^−1^, and PI exhibited a strong linear correlation within the range of 0–800 µg mL^−1^. The linear equation of the standard curve had a correlation coefficient (*R* ^2^) of 0.9967.

### Combining IRE and PI for In Vivo Tumor Treatment–Animal

Seven‐week‐old B6‐hPD1 mice (strain number: T003095) were obtained from GemPharmatech (Nanjing, China). After a week of acclimatization, all tests were performed. The mice were kept in a diurnal cycle for 12 h at 20–24 °C with 44.5%–51.8% humidity. All animal experiments were performed following the protocols approved by the Institutional Animal Care and Use Committee of Zhejiang University School of Medicine (ETHICS CODE ZJU20220530).

### Cell Cultures

The Hepa1‐6 cell line, derived from murine liver cancer, was acquired from the cell repository of the Chinese Academy of Sciences in Shanghai, China, and cultured in DMEM/high glucose (Gibco) supplemented with 10% FBS (Gibco), penicillin (100 U mL^−1^), and streptomycin (100 mg mL^−1^) (HyClone).

### Model Establishment and Treatment Strategy

Various methods for in vivo tumor treatment are illustrated in Figure S6 (Supporting Information). B6‐hPD1 mice were injected with Hepa1‐6 cells (1 × 10^6^ cells) subcutaneously on the right hind limb to establish tumor control. During the 14‐day therapy period, the tumor size and body weight were measured every two days. Homozygous mice were randomly divided into six groups: 1) The control group (*n* = 5) without any treatment; 2) the IRE treatment group (*n* = 5); 3) the PI group (*n* = 5) receiving tail vein injection (5 mg k^−1 ^g, iv, qod × 7); 4) the IRE combined with PI treatment group (*n* = 5) receiving tail vein injection (5 mg k^−1 ^g, iv, qod × 7); 5) the PDA‐CV@PI treatment group (*n* = 5) receiving gavage administration; and 6) the IRE combined with PDA‐CV@PI treatment group (*n* = 5), which was administered by gavage (77 mg k^−1 ^g, ig, qod × 7). The mice were anesthetized using 2% isoflurane with an oxygen flow rate of 3 L min^−1^ and administered a subcutaneous injection of buprenorphine analgesic (0.1 mg k^−1 ^g). IRE was performed on anesthetized rodents by using a dual‐needle array electrode from medical‐grade stainless steel. The tumor was treated with IRE by inserting two needle electrodes spaced 3 mm apart, as depicted in the accompanying illustration. IRE was performed by puncturing the skin above the subcutaneous tumor, encircling the tumor with a two‐needle probe, and administering electrical pulses using a safety foot pedal once the tumor had grown to a diameter of 5 mm. Moreover, IRE was performed under the specified conditions (voltage of 1000 V, pulse duration of 100 µs, and 90 pulses). The drug was administered orally or intravenously following IRE according to a pre‐planned schedule.

### Establishment and Grouping of Orthotopic Transplantation Tumor Model of HCC in Mice

After anesthetizing the B6‐hPD1 mice in each group, the abdominal hair was removed using a shaving machine, and the abdominal skin preparation site was disinfected sequentially with iodophor and 75% ethanol. A longitudinal incision of ≈1.5–2.0 cm was made along the midline of the abdomen from the xiphoid process downward using ophthalmic scissors to expose the xiphoid process. A 2 cm diameter gauze tube was placed on the upper edge of the liver to fully expose it, and the anatomical structure of the liver was confirmed to be normal. A portion of the left lobe of the liver was gently removed using an alcohol swab, and 50 µL of 1–6 hepatic cytosol (2 × 10^7^ mL^−1^) was injected into the hepatic membrane. Most lobes in the injected area displayed a rapid color change, becoming lighter (white spots) or turbid and translucent vacuoles. After injection, the needle was left in place for 1 min and then slowly withdrawn from the white spots or vacuoles. The needle was held in place for 5 min after withdrawing from the area before withdrawing the entire needle. After gently wiping the surface of the liver lobe with a cotton swab dipped in 75% ethanol, the left lobe was gently moved back into the abdominal cavity. Gentamicin (8 µg µL^−1^, 5 mL kg^−1^) was injected into the peritoneal cavity based on the body weights of the mice. The peritoneum and skin were sutured sequentially using 4–0 nonabsorbable sutures. After suturing, the incision and surrounding skin were wiped with 75% ethanol, and the mice were placed on a heating pad at 35 °C for resuscitation. After complete resuscitation, mice were moved into a feeder box for routine rearing.

When the maximum diameter of the orthotopic tumor reached ≈5 mm, the orthotopic tumor B6‐hPD1 mice were randomly divided into the control, IRE, PI, PI + IRE, PDA‐CV@PI, and IRE+PDA‐CV@PI groups, with eight mice in each group.

### Treatment of Orthotopic Tumor Mice

1. IRE group: Mice were placed in the supine position after complete anesthesia with 2% isoflurane. Alcohol cotton balls were used to disinfect the abdomen and the IRE needles. The voltage was set to 1000 V, and the pulse duration was set to 100 µs with 90 pulses. After setting the instrument (Figure S7, Supporting Information), double needles (3 mm apart) were inserted into the tumor margin area in the direction of the longitudinal axis of the tumor under ultrasound guidance to ensure that the target tumor reached incomplete ablation. After ablation, the mice were placed back in their cages and kept warm.
2. PI, PDA‐CV@PI, IRE + PI, and IRE + PDA‐CV@PI groups: The PI group was administered by tail vein injection (5 mg k^−1 ^g, iv, qod × 7), and the PDA‐CV@PI group was administered by gavage (77 mg k^−1 ^g, ig, qod × 7). The IRE + PI (5 mg k^−1 ^g, iv, qod × 7) and IRE + PDA‐CV@PI (77 mg k^−1 ^g, ig, qod × 7) groups were administered 24 h after IRE treatment. Tail vein injection of PBS (2 mL kg^−1^, iv, qod × 7) was administered to the control and IRE groups.
3. The general condition of the mice was observed every two days after treatment, and orthotopic tumor changes were recorded using ultrasound.

### In Vivo MRI Evaluation

MRI experiments were conducted on a 3.0 T MRI Clinical Scanner (General Electric Company, USA) using an MRI wrist coil equipped with this device to facilitate in vivo image acquisition in each group of mice. Mice were immobilized using anesthesia (1% pentobarbital sodium) and positioned in the wrist coil to ensure that the center of the tumor was aligned with the coil center. MRI scans were then performed to capture images of the tumor area using the vertical axis position of the T2 sequence. ImageJ analysis was used to compare the signal strength of the tumor region and tumor dimensions within each group.

### Regional Blood Flow Velocity and Perfused Blood Vessel Distribution

Laser speckle contrast imaging (RFLSI Pro; RWD) was used to detect the real‐time regional velocity and distribution of blood flow and perfused blood vessels of the sciatic nerve and foot pads after anesthesia with isoflurane (RWD, Shenzhen, China). The ImageJ software was used to analyze the blood perfusion areas of the sciatic nerve and foot pads.

### Tumor Size Assessment

An electronic Vernier caliper was used to measure the longest (L) and shortest (S) diameters of the tumor on the right side every two days. Tumor volume was calculated using the formula (L × S^2^)/2. To evaluate tumor effectiveness, the tumor volume on day 0 was compared with the most recent measurement of the tumor volume. The mice were euthanized when the tumor size exceeded 2000 mm^3^, as defined by the Animal Welfare Organization.

### Tumor Tissue Staining and Pathological Assessment

After treatment, five mice were randomly selected from each group and underwent tumor tissue removal using the eyeball method, which involved eliminating peripheral blood. The maximum diameter of the tumor tissue specimens was measured. The tumor specimens were preserved in neutral paraformaldehyde, embedded in paraffin, and sliced to examine the histomorphological changes in the tumor tissue among the different groups. Tissue sections were stained with H&E and observed at various magnifications using a microscope. Furthermore, immunohistochemical staining was performed using anti‐CD8 (dilution: 1:300, GB114196‐100; Servicebio), anti‐CD31 (dilution: 1:300, GB11063‐2; Servicebio) and ki67 (dilution: 1:300, GB111141; Servicebio) antibodies. CD8 was used to demonstrate cytotoxic T‐cell infiltration in the tumor area, Ki67 was used to evaluate tumor cell proliferation, and CD31 was used to analyze the number of tumor vessels. Tumor tissues were examined under a microscope, and images were captured using a 200× magnification lens (200× images for counting and presentation). The tumor tissue was examined for positive staining using the Image‐Pro Plus software (version 6.0; Media Cybernetics Inc., Rockville, MD, USA). The counts were subsequently averaged and subjected to statistical analysis.

### Flow Cytometry Analysis

The tumor on the right side was surgically excised and mechanically dissected using surgical scissors. The cells were then subjected to enzymatic digestion for 1 h at 37 °C in DMEM containing collagenase type IV (1 mg mL^−1^, Sigma), hyaluronidase (1 mg mL^−1^, Sigma), and DNase I (20 U mL^−1^, Sigma). After complete digestion, the cells were filtered through a membrane and resuspended in Hank's solution containing 1% FBS. Erythrocytes were lysed in erythrocyte lysis buffer (Sigma). Anti‐CD45 (Percp/cy 5.5, 1:300), CD25 (FITC, 1:300), CD3 (Brilliant Violet 605, 1:150), CD4 (Brilliant Violet 785, 1:150), CD8 (APC, 1:100), Foxp3 (Brilliant Violet 421, 1:300), PD‐1 (PE, 1:100), CD11c (APC,1:300), CD80 (BV421, 1:300), CD86 (Percp‐cy5.5, 1:300), Ifnγ (PE‐cy7, 1:300), granzyme (PE, 1:300), and NK1.1 (Percp‐cy5.5, 1:300) antibodies were obtained from BioLegend and Becton Dickinson. The cells were then stained using an experimental procedure. The examination was conducted using a BD FACSAria flow cytometer (BD Biosciences, San Jose, CA). Data analysis was performed using the FlowJo software.

### Western Blot (WB)

WB analysis was used to determine the protein levels of semi‐quantitative biomarkers. A 10% sodium dodecyl sulfate‐polyacrylamide gel electrophoresis gel was used for electrophoresis, and the proteins were subsequently transferred to activated nitrocellulose membranes. The membranes were incubated overnight at 4 °C with different primary antibodies, including anti‐PD1 Rabbit pAb (GB11338; Servicebio), anti‐STAT3 Rabbit pAb (GB11176; Servicebio), anti‐Phospho‐STAT3 Rabbit pAb (GB13461; Servicebio), anti‐JAK1 Rabbit mAb (#3344, Cell Signaling Technology), anti‐Phospho‐JAK1 Rabbit mAb (#74129, Cell Signaling Technology), and anti‐beta Actin Rabbit pAb (GB11001; Servicebio). The cells were then incubated with secondary antibodies at room temperature for 1 h. An enhanced chemiluminescence (ECL) kit (Amersham, Piscataway, NJ, USA) was used to visualize the antigen–antibody complexes. Densitometric analysis was used to quantify protein bands.

### Biosafety Test

Following consistent feeding, 12 male B6‐hPD1 mice weighing 20 ± 2 g were randomly divided into 3 groups, each consisting of 6 mice. There were three groups: PDA‐coated CV@PI oral group (80 mg k^−1 ^g), intravenous group with PI (10 mg k^−1 ^g), and normal control group (10 mL kg^−1^ of saline). After a 14‐day administration period, the mice were euthanized, and the blood, liver, heart, spleen, lungs, kidneys, small intestines, and skin were collected. Blood serum and whole blood were collected for biochemical and routine blood tests, respectively, including measurements of AST, ALT, blood urea nitrogen, creatinine, WBC, RBC, HGB, and PCT, among others. H&E staining was performed on tissue sections to enhance the contrast between the structural components of tissue cells to facilitate the visualization of the experimental findings.

### Gut Microbiota Analysis

After 14 days of treatment, tumor‐bearing mice in each group were deprived of food and water the night before euthanasia. On the second day, mice were euthanized by cervical dislocation. The mice were then dissected on ice, and the entire colon was excised using sterile scissors. The intestinal canal was dissected longitudinally, and feces were collected using sterile Eppendorf tubes and stored in a refrigerator at −80 °C. Detection of mouse fecal samples by 16S rRNA gene sequencing was performed by Beijing Kengke Biotechnology Co. Ltd., and MEGA software was used to calculate the diversity (Shannon) and abundance (Chao) indices of the flora.

### Statistical Analysis

Kinetica software (version 4.4.1; Thermo Fisher Scientific Inc., MA, USA) was used to analyze the plasma drug concentration–time data and determine the PK parameters. These parameters were calculated based on Akaike's Information Criteria and goodness of fit obtained from a two‐compartment model. All results are expressed as the mean ± standard deviation (SD). The groups were tested for significant differences using Student's *t*‐test, with a significance level set at *p* < 0.05.

## Conflict of Interest

The authors declare no conflict of interest.

## Author Contributions

C.Z., S.H., and J.Z. contributed equally to this work. C.Z., M.Z., and Z.T. conceived and coordinated the study. C.Z., S.H., J.Z., M.Z., and Z.T. conceived and designed the experiments. C.Z., S.H., J.Z., T.Z., J.C., A.J., and L.S. performed or analyzed the experiments. C.Z., S.H., M.Z., and Z.T. wrote the manuscript.

## Supporting information



Supporting Information

## Data Availability

The data that support the findings of this study are available from the corresponding author upon reasonable request.

## References

[1] H. Sung , J. Ferlay , R. L. Siegel , M. Laversanne , I. Soerjomataram , A. Jemal , F. Bray , CA‐Cancer J. Clin. 2021, 71, 209. 33538338 10.3322/caac.21660

[2] A. Vogel , T. Meyer , G. Sapisochin , R. Salem , A. Saborowski , Lancet 2022, 400, 1345. 36084663 10.1016/S0140-6736(22)01200-4

[3] S. Lun , Y. Yang , Y. Li , Y. Li , B. Zhang , R. Shi , Cancer Lett. 2023, 574, 216334. 37574184 10.1016/j.canlet.2023.216334

[4] D. Liu , T. Song , Biosci. Trends 2021, 15, 142. 33716267 10.5582/bst.2021.01083

[5] A. Forner , M. Reig , J. Bruix , Lancet 2018, 391, 1301. 29307467 10.1016/S0140-6736(18)30010-2

[6] E. Cho , H. A. Cho , C. H. Jun , H. J. Kim , S. B. Cho , S. K. Choi , In Vivo 2019, 33, 1411. 31471386 10.21873/invivo.11618 PMC6755010

[7] L. Kulik , J. K. Heimbach , F. Zaiem , J. Almasri , L. J. Prokop , Z. Wang , M. H. Murad , K. Mohammed , Hepatology 2018, 67, 381. 28859222 10.1002/hep.29485

[8] J. M. Llovet , T. De Baere , L. Kulik , P. K. Haber , T. F. Greten , T. Meyer , R. Lencioni , Nat. Rev. Gastroenterol. Hepatol. 2021, 18, 293. 33510460 10.1038/s41575-020-00395-0

[9] K. Wang , C. Wang , H. Jiang , Y. Zhang , W. Lin , J. Mo , C. Jin , Front. Immunol. 2021, 12, 792781. 34975896 10.3389/fimmu.2021.792781 PMC8714655

[10] M. Yu , S. Li , Eur. J. Surg. Oncol. 2022, 48, 1321. 35012834 10.1016/j.ejso.2021.12.015

[11] A. Eresen , J. Yang , A. Scotti , K. Cai , V. Yaghmai , Z. Zhang , Ann. Transl. Med. 2021, 9, 1089. 34423001 10.21037/atm-21-539 PMC8339821

[12] L. Tian , L. Wang , Y. Qiao , L. Lu , P. Lee , A. Chang , S. Ravi , T. A. Rogers , M. P. Melancon , Molecules 2019, 24, 3560. 31581445 10.3390/molecules24193560 PMC6804038

[13] W. Bäumler , L. P. Beyer , L. Lürken , P. Wiggermann , C. Stroszczynski , M. Dollinger , A. Schicho , Diagnostics 2022, 12, 986. 35454034 10.3390/diagnostics12040986 PMC9026630

[14] R. Lencioni , L. Crocetti , Recent Results Cancer Res. 2013, 190, 181. 22941021 10.1007/978-3-642-16037-0_12

[15] J. Yu , X. L. Yu , Z. Y. Han , Z. G. Cheng , F. Y. Liu , H. Y. Zhai , M. J. Mu , Y. M. Liu , P. Liang , Gut 2017, 66, 1172. 27884919 10.1136/gutjnl-2016-312629 PMC5532455

[16] D. S. Lu , S. T. Kee , E. W. Lee , Tech. Vasc. Interventional Radiol. 2013, 16, 277. 10.1053/j.tvir.2013.08.010 24238383

[17] L. Mannelli , S. A. Padia , R. S. Yeung , D. E. Green , Liver Int. 2013, 33, 104. 22925039 10.1111/liv.12000

[18] Z. Xu , X. Wang , H. Ke , G. Lyu , Cryobiology 2023, 112, 104560. 37499964 10.1016/j.cryobiol.2023.104560

[19] M. Kudo , Liver Cancer 2019, 8, 221. 31602367 10.1159/000501501 PMC6738201

[20] V. Verma , R. K. Shrimali , S. Ahmad , W. Dai , H. Wang , S. Lu , R. Nandre , P. Gaur , J. Lopez , M. Sade‐Feldman , K. Yizhak , S. L. Bjorgaard , K. T. Flaherty , J. A. Wargo , G. M. Boland , R. J. Sullivan , G. Getz , S. A. Hammond , M. Tan , J. Qi , P. Wong , T. Merghoub , J. Wolchok , N. Hacohen , J. E. Janik , M. Mkrtichyan , S. Gupta , S. N. Khleif , Nat. Immunol. 2019, 20, 1231. 31358999 10.1038/s41590-019-0441-y PMC7472661

[21] P. S. Hegde , D. S. Chen , Immunity 2020, 52, 17. 31940268 10.1016/j.immuni.2019.12.011

[22] X. Guo , F. Du , Q. Liu , Y. Guo , Q. Wang , W. Huang , Z. Wang , X. Ding , Z. Wu , BMC Cancer 2021, 21, 443. 33882892 10.1186/s12885-021-08176-x PMC8061072

[23] A. L. Cheng , C. Hsu , S. L. Chan , S. P. Choo , M. Kudo , J. Hepatol. 2020, 72, 307. 31954494 10.1016/j.jhep.2019.09.025

[24] Y. Yu , S. Wang , N. Su , S. Pan , B. Tu , J. Zhao , Y. Shen , Q. Qiu , X. Liu , J. Luan , F. S. Wang , F. Meng , M. Shi , Front. Oncol. 2022, 12, 906824. 35756643 10.3389/fonc.2022.906824 PMC9232255

[25] X. Yang , W. Li , S. Li , S. Chen , Z. Hu , Z. He , X. Zhu , X. Niu , X. Zhou , H. Li , Y. Xiao , J. Liu , X. Sui , G. Chen , Y. Gao , J. Controlled Release 2024, 365, 654. 10.1016/j.jconrel.2023.11.042 38030081

[26] S. Paul , S. Bhuyan , D. D. Balasoupramanien , A. Palaniappan , ACS Omega 2024, 9, 24121. 38882129 10.1021/acsomega.3c10305 PMC11170654

[27] G. Chen , W. Kang , W. Li , S. Chen , Y. Gao , Theranostics 2022, 12, 1419. 35154498 10.7150/thno.61747 PMC8771547

[28] W. Li , X. Zhu , X. Zhou , X. Wang , W. Zhai , B. Li , J. Du , G. Li , X. Sui , Y. Wu , M. Zhai , Y. Qi , G. Chen , Y. Gao , J. Controlled Release 2021, 334, 376. 10.1016/j.jconrel.2021.04.036 33940058

[29] P. G. Sasikumar , N. S. Sudarshan , S. Adurthi , R. K. Ramachandra , D. S. Samiulla , A. Lakshminarasimhan , A. Ramanathan , T. Chandrasekhar , A. A. Dhudashiya , S. R. Talapati , N. Gowda , S. Palakolanu , J. Mani , B. Srinivasrao , D. Joseph , N. Kumar , R. Nair , H. S. Atreya , N. Gowda , M. Ramachandra , Commun. Biol. 2021, 4, 699. 34103659 10.1038/s42003-021-02191-1 PMC8187357

[30] S. Poudwal , A. Misra , P. Shende , J. Drug Targeting 2021, 29, 834. 10.1080/1061186X.2021.1894434 33620269

[31] H. Wu , T. Guo , J. Nan , L. Yang , G. Liao , H. J. Park , J. Li , Macromol. Biosci. 2022, 22, e2100493. 35182103 10.1002/mabi.202100493

[32] E. Muntoni , E. Marini , N. Ahmadi , P. Milla , C. Ghè , A. Bargoni , M. T. Capucchio , E. Biasibetti , L. Battaglia , Acta Diabetol. 2019, 56, 1283. 31407113 10.1007/s00592-019-01403-9

[33] F. Mainini , M. R. Eccles , Molecules 2020, 25, 2692. 32532030 10.3390/molecules25112692 PMC7321291

[34] F. Haghiralsadat , G. Amoabediny , S. Naderinezhad , T. Forouzanfar , M. N. Helder , B. Zandieh‐Doulabi , Artif. Cells Nanomed. Biotechnol. 2018, 46, 684. 29475393 10.1080/21691401.2018.1434533

[35] D. W. Malcolm , J. J. Varghese , J. E. Sorrells , C. E. Ovitt , D. S. W. Benoit , ACS Appl. Nano Mater. 2018, 12, 187. 10.1021/acsnano.7b05528 PMC5987762 29232104

[36] E. R. Soto , H. C. Kim , H. Yagita , M. De Jesus , G. R. Ostroff , ACS Appl. Bio. Mater. 2019, 2, 3748. 10.1021/acsabm.9b00379 35021348

[37] Z. Máté , E. Horváth , G. Kozma , T. Simon , Z. Kónya , E. Paulik , A. Papp , A. Szabó , Biol. Trace Elem. Res. 2016, 171, 156. 26384687 10.1007/s12011-015-0508-z

[38] R. Bartucci , A. Paramanandana , Y. L. Boersma , P. Olinga , A. Salvati , Nanotoxicology 2020, 14, 847. 32536243 10.1080/17435390.2020.1771785

[39] U. Neumann , F. Derwenskus , A. Gille , S. Louis , U. Schmid‐Staiger , K. Briviba , S. C. Bischoff , Nutrients 2018, 10, 965. 30049974 10.3390/nu10080965 PMC6116023

[40] A. A. Mohamed , K. M. E. Bohy , G. G. Moustafa , H. H. Mohammed , M. M. M. Metwally , H. E. D. Mohammed , M. A. Nassan , T. M. Saber , Biology 2022, 11, 279. 35205143 10.3390/biology11020279 PMC8869302

[41] D. Zhong , D. Zhang , T. Xie , M. Zhou , Small 2020, 16, e2000819. 32297465 10.1002/smll.202000819

[42] D. Zhong , D. Zhang , W. Chen , J. He , C. Ren , X. Zhang , N. Kong , W. Tao , M. Zhou , Sci. Adv. 2021, 7, eabi9265. 34818040 10.1126/sciadv.abi9265 PMC8612690

[43] D. Zhong , W. Li , S. Hua , Y. Qi , T. Xie , Y. Qiao , M. Zhou , Theranostics 2021, 11, 3580. 33664849 10.7150/thno.55441 PMC7914342

[44] A. Jin , Y. Wang , K. Lin , L. Jiang , Bioact. Mater. 2020, 5, 522. 32322763 10.1016/j.bioactmat.2020.04.003 PMC7170807

[45] Z. Du , Y. Mao , P. Zhang , J. Hu , J. Fu , Q. You , J. Yin , ACS Appl. Mater. Interfaces 2021, 13, 35518. 34286569 10.1021/acsami.1c09610

[46] Y. Bian , H. Wang , J. Xu , Z. Wang , X. Du , Y. Wang , Y. Du , Biomed. Mater. 2021, 16, 025003. 33470977 10.1088/1748-605X/abdd6f

[47] B. Wang , T. Yuan , L. Zha , Y. Liu , W. Chen , C. Zhang , Y. Bao , Q. Dong , Mol. Pharmaceutics 2021, 18, 1470. 10.1021/acs.molpharmaceut.1c00030 33586444

[48] A. Jabbar , K. Rehman , T. Jabri , T. Kanwal , S. Perveen , M. A. Rashid , M. Kazi , S. Ahmad Khan , S. Saifullah , M. R. Shah , Drug Delivery 2023, 30, 2159587. 36718806 10.1080/10717544.2022.2159587 PMC9891165

[49] M. Ramos‐Casals , J. R. Brahmer , M. K. Callahan , A. Flores‐Chávez , N. Keegan , M. A. Khamashta , O. Lambotte , X. Marlette , A. Prat , M. E. Suarez‐Almazor , Nat. Rev. Dis. Primers 2020, 6, 38. 32382051 10.1038/s41572-020-0160-6 PMC9728094

[50] J. Huang , H. N. Mo , D. W. Wu , X. L. Chen , L. Y. Ma , B. Lan , D. Qu , Q. Yang , B. H. Xu , J. Clin. Oncol. 2017, 35, 15.

[51] T. Wada , K. Sugimoto , K. Sakamaki , H. Takahashi , T. Kakegawa , Y. Tomita , M. Abe , Y. Yoshimasu , H. Takeuchi , T. Itoi , Cancers 2023, 15, 732. 36765689 10.3390/cancers15030732 PMC9913859

[52] F. Ridouani , M. Ghosn , F. Cornelis , E. N. Petre , M. Hsu , C. S. Moskowitz , P. T. Kingham , S. B. Solomon , G. Srimathveeravalli , Medicina 2021, 57, 877. 34577800 10.3390/medicina57090877 PMC8467214

[53] Q. Shao , S. O'Flanagan , T. Lam , P. Roy , F. Pelaez , B. J. Burbach , S. M. Azarin , Y. Shimizu , J. C. Bischof , Int. J. Hyperthermia 2019, 36, 130. 30676126 10.1080/02656736.2018.1539253

[54] F. Babikr , J. Wan , A. Xu , Z. Wu , S. Ahmed , A. Freywald , R. Chibbar , Y. Wu , M. Moser , G. Groot , W. Zhang , B. Zhang , J. Xiang , Cell. Mol. Immunol. 2021, 18, 2632. 34782757 10.1038/s41423-021-00796-4 PMC8633376

[55] A. José , L. Sobrevals , A. Ivorra , C. Fillat , Cancer Lett. 2012, 317, 16. 22079741 10.1016/j.canlet.2011.11.004

[56] S. B. White , Z. Zhang , J. Chen , V. R. Gogineni , A. C. Larson , J. Vasc. Interventional Radiol. 2018, 29, 1764. 10.1016/j.jvir.2018.07.009 30316676

[57] C. He , X. Huang , Y. Zhang , X. Lin , S. Li , Clin. Transl. Med. 2020, 10, e39. 32508058 10.1002/ctm2.39 PMC7403705

[58] V. M. Ringel‐Scaia , N. Beitel‐White , M. F. Lorenzo , R. M. Brock , K. E. Huie , S. Coutermarsh‐Ott , K. Eden , D. K. McDaniel , S. S. Verbridge , J. H. Rossmeisl , K. J. Oestreich , R. V. Davalos , I. C. Allen , EBioMedicine 2019, 44, 112. 31130474 10.1016/j.ebiom.2019.05.036 PMC6606957

[59] Z. Dai , Z. Wang , K. Lei , J. Liao , Z. Peng , M. Lin , P. Liang , J. Yu , S. Peng , S. Chen , M. Kuang , Cancer Lett. 2021, 503, 1. 33444692 10.1016/j.canlet.2021.01.001

[60] F. Wang , C. Xu , G. Li , P. Lv , J. Gu , Exp. Cell. Res. 2021, 409, 112910. 34801560 10.1016/j.yexcr.2021.112910

[61] H. Pandit , Y. K. Hong , Y. Li , J. Rostas , Z. Pulliam , S. P. Li , R. C. G. Martin , Ann. Surg. Oncol. 2019, 26, 800. 10.1245/s10434-018-07144-3 30610562

[62] J. Zhao , X. Wen , L. Tian , T. Li , C. Xu , X. Wen , M. P. Melancon , S. Gupta , B. Shen , W. Peng , C. Li , Nat. Commun. 2019, 10, 899. 30796212 10.1038/s41467-019-08782-1 PMC6385305

[63] P. Philips , D. Hays , R. C. Martin , PLoS One 2013, 8, e76260. 24223700 10.1371/journal.pone.0076260 PMC3815199

[64] H. Van Damme , B. Dombrecht , M. Kiss , H. Roose , E. Allen , E. Van Overmeire , D. Kancheva , L. Martens , A. Murgaski , P. M. R. Bardet , G. Blancke , M. Jans , E. Bolli , M. S. Martins , Y. Elkrim , J. Dooley , L. Boon , J. K. Schwarze , F. Tacke , K. Movahedi , N. Vandamme , B. Neyns , S. Ocak , I. Scheyltjens , L. Vereecke , F. A. Nana , P. Merchiers , D. Laoui , J. A. Van Ginderachter , J. Immunother. Cancer 2021, 9, e001749. 33589525 10.1136/jitc-2020-001749 PMC7887378

[65] R. Wang , H. Liu , P. He , D. An , X. Guo , X. Zhang , M. Feng , Front. Immunol. 2022, 13, 947756. 36003387 10.3389/fimmu.2022.947756 PMC9393481

[66] V. Rigo , L. Emionite , A. Daga , S. Astigiano , M. V. Corrias , C. Quintarelli , F. Locatelli , S. Ferrini , M. Croce , Sci. Rep. 2017, 7, 14049. 29070883 10.1038/s41598-017-14417-6 PMC5656588

[67] S. Qin , Z. Ren , Z. Meng , Z. Chen , X. Chai , J. Xiong , Y. Bai , L. Yang , H. Zhu , W. Fang , X. Lin , X. Chen , E. Li , L. Wang , C. Chen , J. Zou , Lancet Oncol. 2020, 21, 571. 32112738 10.1016/S1470-2045(20)30011-5

[68] D. S. Mandlik , S. K. Mandlik , H. B. Choudhary , World J. Gastroenterol. 2023, 29, 1054. 36844141 10.3748/wjg.v29.i6.1054 PMC9950866

[69] K. C. Kwon , D. Verma , N. D. Singh , R. Herzog , H. Daniell , Adv. Drug. Delivery Rev. 2013, 65, 782. 10.1016/j.addr.2012.10.005 PMC3582797 23099275

[70] S. Rosales‐Mendoza , R. Nieto‐Gómez , Trends Biotechnol. 2018, 36, 1054. 29980327 10.1016/j.tibtech.2018.05.010

[71] Y. Torres‐Tiji , F. J. Fields , S. P. Mayfield , Biotechnol. Adv. 2020, 41, 107536. 32194145 10.1016/j.biotechadv.2020.107536

[72] F. Khavari , M. Saidijam , M. Taheri , F. Nouri , Mol. Biol. Rep. 2021, 48, 4757. 34028654 10.1007/s11033-021-06422-w PMC8142882

[73] D. Yuan , Q. Zhao , S. Yan , S. Y. Tang , Y. Zhang , G. Yun , N. T. Nguyen , J. Zhang , M. Li , W. Li , Lab Chip 2019, 19, 2811. 31312819 10.1039/c9lc00482c

[74] B. Chénais , Molecules 2021, 26, 1185. 33672160 10.3390/molecules26041185 PMC7926806

[75] X. Yan , J. Xu , Q. Zhou , D. Jin , C. I. Vong , Q. Feng , D. H. L. Ng , L. Bian , L. Zhang , Appl. Mater. Today 2019, 15, 242.

[76] D. H. Miller , D. T. Lamport , M. Miller , Science 1972, 176, 918. 5033634 10.1126/science.176.4037.918

[77] D. B. Weibel , P. Garstecki , D. Ryan , W. R. DiLuzio , M. Mayer , J. E. Seto , G. M. Whitesides , Proc. Natl. Acad. Sci. U. S. A. 2005, 102, 11963. 16103369 10.1073/pnas.0505481102 PMC1189341

[78] K. Ma , S. Chen , Y. Wu , Y. Ma , H. Qiao , J. Fan , H. Wu , Appl. Microbiol. Biotechnol. 2022, 106, 773 34989826 10.1007/s00253-021-11751-8

[79] B. Reis , L. Ramos‐Pinto , S. A. Cunha , M. Pintado , J. L. da Silva , J. Dias , L. Conceição , E. Matos , B. Costas , Mar. Drugs 2022, 20, 407. 35877700 10.3390/md20070407 PMC9323325

[80] J. Park , T. F. Brust , H. J. Lee , S. C. Lee , V. J. Watts , Y. Yeo , ACS Nano 2014, 8, 3347. 24628245 10.1021/nn405809c PMC4107448

[81] H. Lee , S. M. Dellatore , W. M. Miller , P. B. Messersmith , Science 2007, 318, 426. 17947576 10.1126/science.1147241 PMC2601629

[82] W. Cheng , X. Zeng , H. Chen , Z. Li , W. Zeng , L. Mei , Y. Zhao , ACS Nano 2019, 13, 8537. 31369230 10.1021/acsnano.9b04436

[83] I. Milosevic , A. Vujovic , A. Barac , M. Djelic , M. Korac , A. Radovanovic Spurnic , I. Gmizic , O. Stevanovic , V. Djordjevic , N. Lekic , E. Russo , A. Amedei , Int. J. Mol. Sci. 2019, 20, 395. 30658519 10.3390/ijms20020395 PMC6358912

[84] L. Shi , J. Sheng , G. Chen , P. Zhu , C. Shi , B. Li , C. Park , J. Wang , B. Zhang , Z. Liu , X. Yang , J. Immunother. Cancer 2020, 8, e000973. 33028692 10.1136/jitc-2020-000973 PMC7542661

[85] L. Derosa , B. Routy , A. M. Thomas , V. Iebba , G. Zalcman , S. Friard , J. Mazieres , C. Audigier‐Valette , D. Moro‐Sibilot , F. Goldwasser , C. A. C. Silva , S. Terrisse , M. Bonvalet , A. Scherpereel , H. Pegliasco , C. Richard , F. Ghiringhelli , A. Elkrief , A. Desilets , F. Blanc‐Durand , F. Cumbo , A. Blanco , R. Boidot , S. Chevrier , R. Daillère , G. Kroemer , L. Alla , N. Pons , E. Le Chatelier , N. Galleron , et al., Nat. Med. 2022, 28, 315. 35115705 10.1038/s41591-021-01655-5 PMC9330544

