# Supplementary material for: Oral Microalgae‐Based Biosystem to Enhance Irreversible Electroporation Immunotherapy in Hepatocellular Carcinoma
Source: Adv Sci (Weinh). 2025 Jan 28;12(15):2409381. doi: 10.1002/advs.202409381 (PMC12005737; doi:10.1002/advs.202409381)
Supplement: Supplementary file 1 — Supporting Information [file ADVS-12-2409381-s001.docx]

Supporting Information

**Oral microalgae-based biosystem to enhance irreversible**

**electroporation immunotherapy in hepatocellular carcinoma**

Cheng Zeng^1,2^, Shiyuan Hua^4,5,6^, Jiayu Zhou^7^, Tangye Zeng^1,2^, Jianke Chen^1^, Lijian Su^1^, Angfeng Jiang^1,2^, Min Zhou^1,4,5,6^*, Zhe Tang^1,2,3^*

C.Z., S.H. and J.Z. contributed equally to this paper.

**Dr. C. Zeng, Mr. T. Zeng, Mr. L. Su, Mr. A. Jiang, Prof.Dr. M. Zhou, Prof. Dr. Z. Tang**

Department of Surgery, Center for Cancer Medicine, the Fourth Affiliated Hospital of School of Medicine, and International School of Medicine, International Institutes of Medicine, Zhejiang University, Yiwu, China, 322000;

E-mail: zhoum@zju.edu.cn (Min Zhou), 8xi@zju.edu.cn (Zhe Tang)

**Mr. S. Hua, Prof. Dr. M. Zhou**

Zhejiang University-University of Edinburgh Institute (ZJU-UoE Institute), Zhejiang University, Haining 314400,China;

**Prof. Dr. Z. Tang**

Department of Surgery, The Second Affiliated Hospital, Zhejiang University School of Medicine, Hangzhou, 310000, China;

**Dr. C. Zeng, Mr. T. Zeng, Mr. A. Jiang, Prof. Dr. Z. Tang**

Zhejiang Key Laboratory of Precision Diagnosis and Treatment for Lung Cancer, Yiwu 322000, China

**Mr. S. Hua, Prof. Dr. M. Zhou**

Institute of Translational Medicine, Zhejiang University, Hangzhou 310029,China;

**Mr. S. Hua, Prof. Dr. M. Zhou**

Zhejiang University-Ordos City Etuoke Banner Joint Research Center, Haining, 314400, China.

**Mr. S. Hua, Prof. Dr. M. Zhou**

The National Key Laboratory of Biobased Transportation Fuel Technology, Zhejiang University, Hangzhou, 310027 China.

**Mr. J. Zhou**

Shihezi University School of Medicine, Shihezi, Xinjiang, 832002，China.

**This file includes the Experimental Section.**

**1.1 Fourier Transform Infrared Spectrometer (FTIR) analysis**

**1.2 Regional blood flow velocity and perfused blood vessel distribution imaging**

**1.3 Immunofluorescence staining**

**1.4 Western blot**

**1.5 TUNEL analysis of HCC *in situ***

**1.6 Therapeutic effects of different treatments in tumor models**

**1.7 Ultrasound-assisted minimally invasive IRE treatment imaging**

**Table S1. Results of release curve fitting.**

**Table S2. Pharmacokinetic parameters of different formulated drugs in mice.**

**Table S3. Hematological parameters of different formulated drugs in mice.**

**Experimental Section**

**1.1 Fourier Transform Infrared Spectrometer (FTIR) analysis**

FTIR analyses of CV, PI, CV@PI, PDA, and PDA-CV@PI were performed using an iS20 infrared spectrophotometer (Thermo, USA). IR spectra were recorded between 500 and 4000 cm^–1^.

As illustrated in Figure S1, both CV and PI exhibited infrared absorption peaks at 3224, 2915, 1634, 1250, and 1055 cm^–1^, which correspond to the stretching vibrations of the O-H, C-H, C=C, and C-O bonds, respectively. Notably, the C-H and C=C stretching vibration peaks at 2915 and 1634 cm^–1^ exhibited equal intensity. Similarly, PI also displayed infrared characteristic peaks for O-H and C=C bonds at 3224 and 1634 cm^–1^; however, these peaks were fewer and weaker than those of CV. When the drug was combined with the carrier, the out-of-plane bending vibration peak of the C-H bond at 860 cm^–1^ in CV disappeared in CV@PI, and the stretching vibration peak intensity of the C-O bond at 1055 cm^–1^ decreased. These observations suggest that PI encapsulated or underwent a subtle reaction with CV during the loading process, leading to the disappearance and weakening of the characteristic peaks, thereby confirming the successful loading of PI into the CV. For PDA, the peaks located at 1077, 1438, 1601, and 3224 cm^–1^ correspond to the stretching vibrations of C-O, C-N, C=C, and O-H bonds, respectively. The consistency of the PDA structure with that reported in the literature indicates that PDA was successfully prepared. Finally, after CV@PI was encapsulated by PDA, the intensities of the various chemical absorption peaks in PDA-CV@PI were significantly reduced. The peaks corresponding to the O-H, C-H, C=C, and C-O bonds were prominent, indicating that PDA has successfully encapsulated CV@PI.


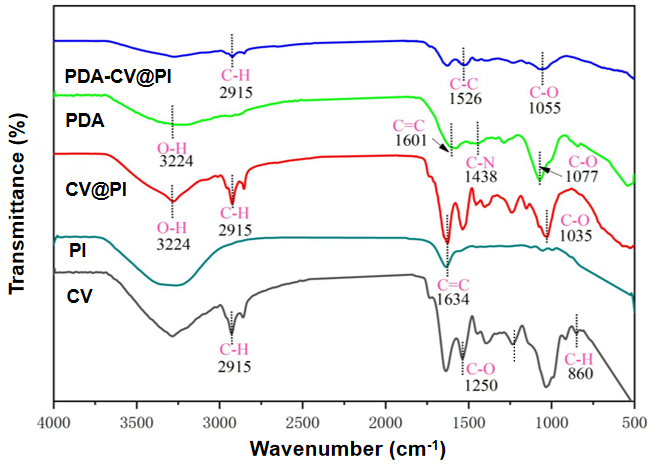


**Figure S1. FTIR spectra of CV, PI, CV@PI, PDA, and PDA-CV@PI.**

**1.2 Regional blood flow velocity and perfused blood vessel distribution imaging**

As depicted in Figure S2, Laser Speckle Contrast Imaging/LSCI (RFLSI Pro; RWD) was used to detect real-time regional velocity and distribution of blood flow and perfused blood vessels of the sciatic nerve and foot pads following anesthesia with isoflurane (RWD, Shenzhen, China). The ImageJ software was used to analyze the blood perfusion areas of the sciatic nerve and foot pads.


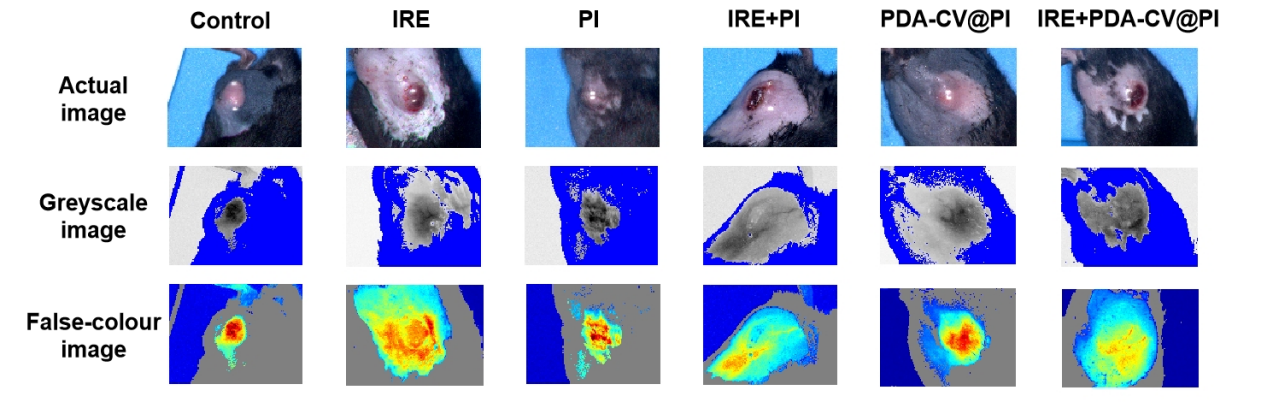


**Figure S2. Distribution of subcutaneous tumor vasculature and vascular perfusion in mice.**

**1.3 Immunofluorescence staining**

Tumor specimens were fixed in neutral paraformaldehyde, embedded in paraffin, and sectioned to examine histomorphological and apoptotic changes in tumor tissues between different groups. The tissue sections were then stained with the TUNEL reaction solution and DAPI and observed at different microscopic magnifications (Figure S3).


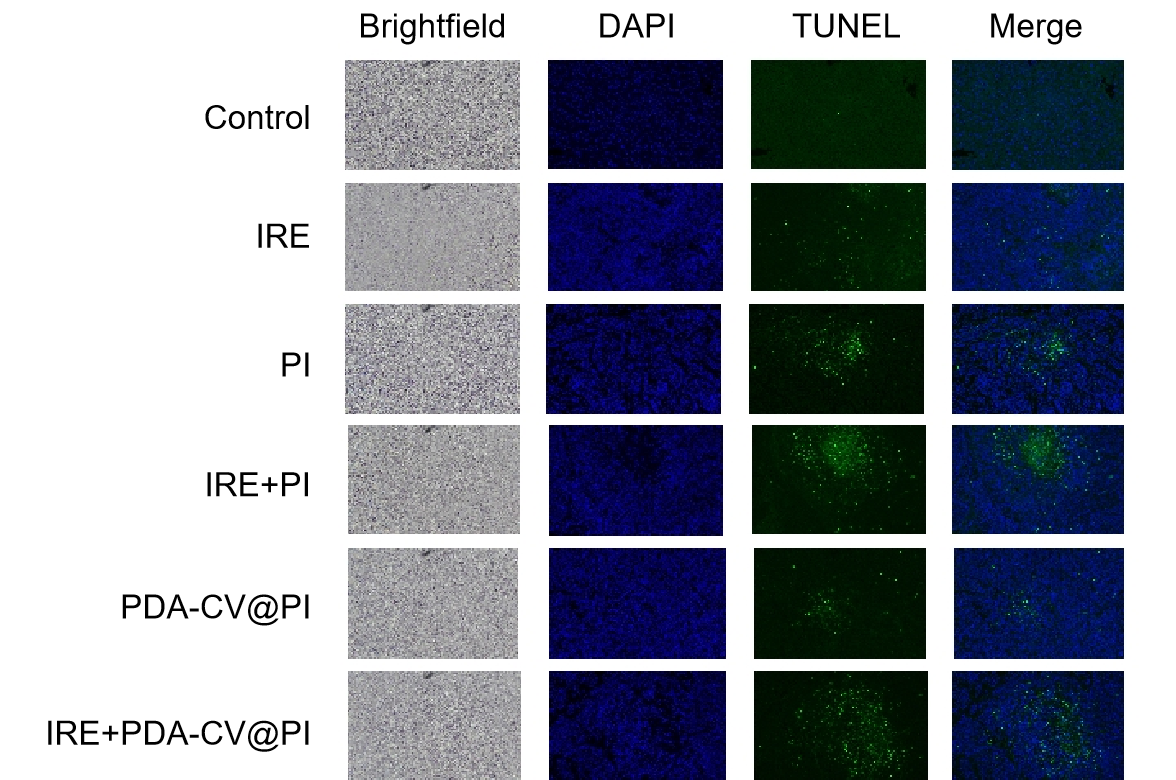


**Figure S3. Apoptosis staining images for each group.**

**1.4 Western blot**

As illustrated in Figure S4, the protein levels of semi-quantitative biomarkers were determined using WB analysis. A 10% SDS-PAGE gel was used for electrophoresis, and the proteins were subsequently transferred to activated nitrocellulose membranes. Different primary antibodies, including anti-PD1 Rabbit pAb (GB11338; Servicebio), anti-STAT3 Rabbit pAb (GB11176; Servicebio), anti-Phospho-STAT3 Rabbit pAb (GB13461; Servicebio), anti-JAK1 Rabbit mAb (#3344, Cell Signaling Technology), anti-Phospho-JAK1 Rabbit mAb (#74129, Cell Signaling Technology), and anti-beta Actin Rabbit pAb (GB11001; Servicebio), were mixed with the membranes. The mixture was then incubated overnight at 4 °C. Subsequently, the mixture was incubated with secondary antibodies at room temperature for 1 h. An ECL kit (Amersham, Piscataway, NJ, USA) was used to visualize the antigen-antibody complexes. Densitometric analysis was used to quantify protein bands.


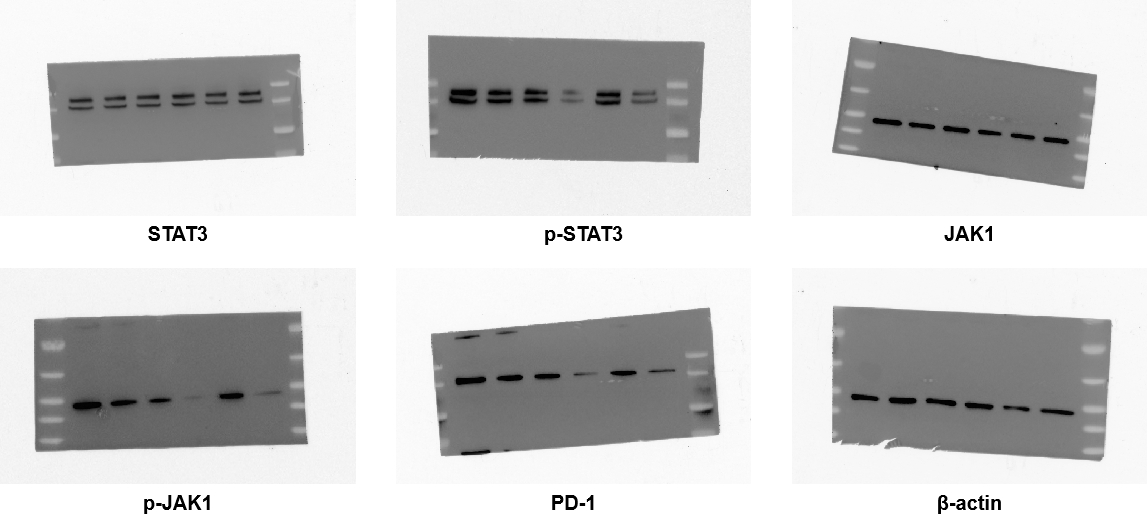


**Figure S4. Original image of the entire WB film.**

**1.5 TUNEL analysis of HCC *in situ***

As displayed in Figure S5, tumor specimens were fixed in neutral paraformaldehyde, embedded in paraffin, and sectioned to examine apoptotic changes in *in situ* HCC tissues across different groups. The tissue sections were then stained with TUNEL reaction solution and DAPI and observed at different microscopic magnifications.


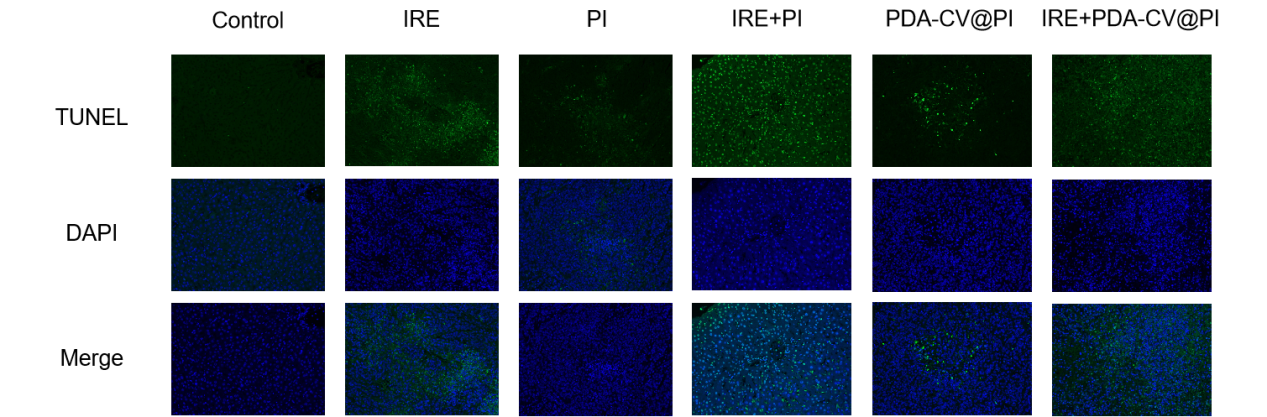


**Figure S5. *In Vivo* TUNEL staining images for each group.**

**1.6 Therapeutic effects of different treatments in tumor models**

As illustrated in Figure S6, during the 14-day treatment period, two groups were designed, including subcutaneous and orthotopic transplantation mouse models, to assess the dynamic changes in tumor growth. Treatments for each group of mice included control, IRE, PI injection, IRE + PI injection, PDA-CV@PI, and IRE + PDA-CV@PI oral.


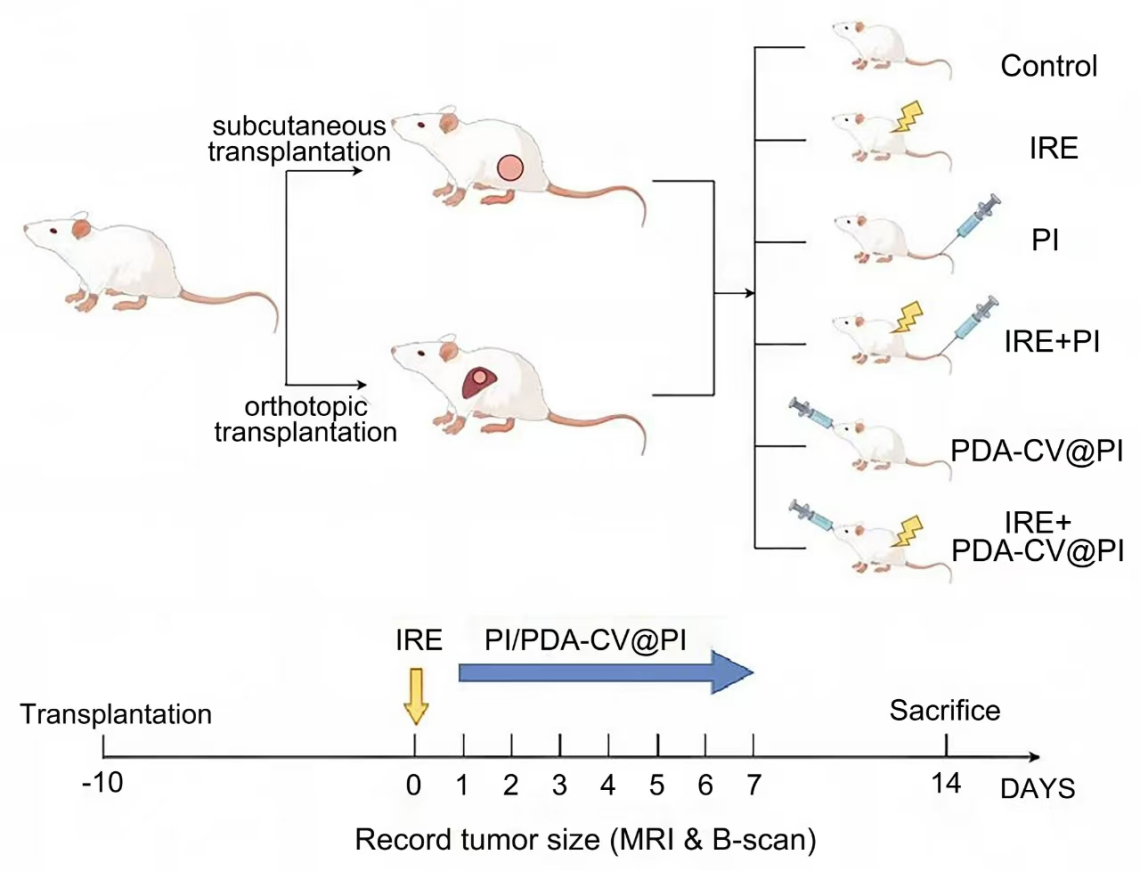


**Figure S6. Schematic diagram of mouse grouping in different tumor models.**

**1.7 Ultrasound-assisted minimally invasive IRE treatment imaging**

B6-hPD1 mice were anesthetized with 2% isoflurane gas. Following complete anesthesia, the mice were placed in a supine position. The abdomen and IRE needles were sterilized with alcohol cotton balls, the voltage was set to 1000 V, and the pulse duration was set to 100 μs, with a total of 90 pulses. As depicted in Figure S7, after the instrument was debugged, double needles (3 mm apart) were inserted into the tumor margin area along the longitudinal axis of the tumor with the assistance of B-scan ultrasonography to ensure that the target tumor reached incomplete ablation.


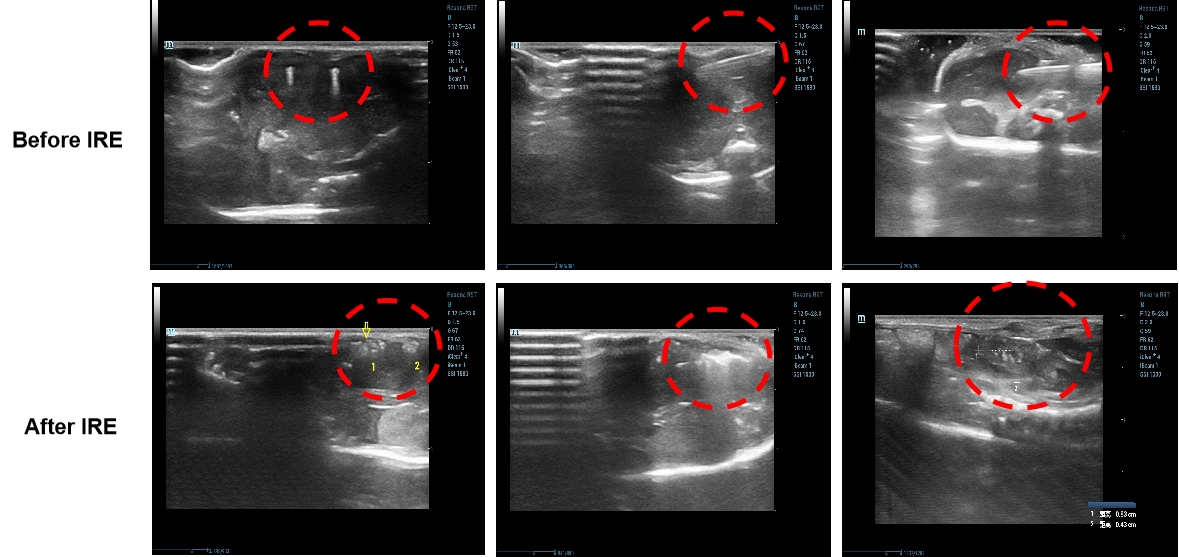


**Figure S7. B-scan Ultrasound-assisted minimally invasive IRE treatment.**

Table S1. Results of release curve fitting.

| Release | A | r^2^ | B | r^2^ |
| --- | --- | --- | --- | --- |
| Zero order | Q=0.468t+57.386 | 0.318 | Q=0.0585t+32.04 | 0.7405 |
| One order | In(1-Q)=-0.04t+3.6524 | 0.9171 | In(1-Q)=-0.005t+4.5118 | 0.9834 |
| Higuchi | Q=7.3549t_1/2_+38.199 | 0.5861 | Q=2.4814t_1/2_+18.468 | 0.9152 |
| Weibull | InIn[1/(1-Q)]=0.3972Int-0.3323 | 0.936 | InIn[1/(1-Q)]=0.553Int-2.5872 | 0.8775 |

**A: CV@PI oral group; B: PDA-CV@PI oral group**

Table S2. Pharmacokinetic parameters of different formulated drugs in mice.

| Parameter | A | B | C |
| --- | --- | --- | --- |
| C_max_ (mg/L) | 215.87 ± 23.13 | 73.48 ± 7.97 | 199.57 ± 32.02**^*^** |
| T_max_ (h) | 19.361 ± 3.81 | 1.83 ± 0.42 | 39.31 ± 6.15**^**^** |
| t 1/2 (h) | 30.79 ± 4.29 | 18.87 ± 3.33 | 53.69 ± 5.51**^*^** |
| AUC (0-∞) (mg/L*h) | 26939.97 ± 2254.87 | 11927.41 ± 1754.79 | 32199.31 ± 3314.03**^*^** |
| MRT (0-∞) (h) | 90.37 ± 14.46 | 47.36 ± 5.03 | 131.95 ± 2.84**^*^** |

**A: PI intravenous group; B: PI oral group; and C: PDA-CV@PI oral group. Values are expressed as mean ± SD (n = 6), ^*^*P* < 0.05, ^**^*P* < 0.01, compared to the PI oral group.**

**Table S3. Hematological parameters of different formulated drugs in mice.**

| Indicator  Group | Control | PI | PDA-CV@PI |
| --- | --- | --- | --- |
| WBC (10^9^/L) | 5.79 ± 0.49 | 14.04 ± 2.95** | 6.26 ± 0.93 |
| RBC (10^12^/L) | 9.95 ± 0.11 | 9.71 ± 0.59 | 9.25 ± 0.42 |
| HGB (g/L) | 137.33 ± 0.58 | 134.33 ± 6.43 | 131.33 ± 3.51 |
| PCT (%) | 0.53 ± 0.15 | 1.14 ± 0.15** | 0.57 ± 0.08 |
| HCT (%) | 42.23 ± 1.06 | 41.47 ± 2.11 | 40.17 ± 1.56 |
| MCV (fL) | 43.10 ± 0.62 | 43.10 ± 0.53 | 43.43 ± 0.29 |
| MCH (pg) | 13.87 ± 0.25 | 13.77 ± 0.23 | 14.20 ± 0.26 |
| MCHC (g/L) | 321.00 ± 4.00 | 319.00 ± 1.00 | 327.33 ± 4.04 |
| PLT (10^9^/L) | 1120.33 ± 175.46 | 1504.00 ± 388.39 | 1156.33 ± 329.20 |

**Values are expressed as mean ± SD (n = 3), **P* < 0.05, ***P* < 0.01, compared to the control group.**
